# An Arabidopsis SUMO E3 Ligase, SIZ1, Negatively Regulates Photomorphogenesis by Promoting COP1 Activity

**DOI:** 10.1371/journal.pgen.1006016

**Published:** 2016-04-29

**Authors:** Xiao-Li Lin, De Niu, Zi-Liang Hu, Dae Heon Kim, Yin Hua Jin, Bin Cai, Peng Liu, Kenji Miura, Dae-Jin Yun, Woe-Yeon Kim, Rongcheng Lin, Jing Bo Jin

**Affiliations:** 1 Key Laboratory of Plant Molecular Physiology, Institute of Botany, Chinese Academy of Sciences, Beijing, China; 2 Department of Biology, Sunchon National University, Sunchon, Republic of Korea; 3 Faculty of Life and Environmental Sciences, University of Tsukuba, Tsukuba, Japan; 4 Division of Applied Life Science (BK21Plus), PMBBRC & IALS, Gyeongsang National University, Jinju, Republic of Korea; 5 Key Laboratory of Photobiology, Institute of Botany, Chinese Academy of Sciences, Beijing, China; Peking University, CHINA

## Abstract

COP1 (CONSTITUTIVE PHOTOMORPHOGENIC 1), a ubiquitin E3 ligase, is a central negative regulator of photomorphogenesis. However, how COP1 activity is regulated by post-translational modifications remains largely unknown. Here we show that SUMO (small ubiquitin-like modifier) modification enhances COP1 activity. Loss-of-function *siz1* mutant seedlings exhibit a weak constitutive photomorphogenic phenotype. SIZ1 physically interacts with COP1 and mediates the sumoylation of COP1. A K193R substitution in COP1 blocks its SUMO modification and reduces COP1 activity *in vitro* and *in planta*. Consistently, COP1 activity is reduced in *siz1* and the level of HY5, a COP1 target protein, is increased in *siz1*. Sumoylated COP1 may exhibits higher transubiquitination activity than does non-sumoylated COP1, but SIZ1-mediated SUMO modification does not affect COP1 dimerization, COP1-HY5 interaction, and nuclear accumulation of COP1. Interestingly, prolonged light exposure reduces the sumoylation level of COP1, and COP1 mediates the ubiquitination and degradation of SIZ1. These regulatory mechanisms may maintain the homeostasis of COP1 activity, ensuing proper photomorphogenic development in changing light environment. Our genetic and biochemical studies identify a function for SIZ1 in photomorphogenesis and reveal a novel SUMO-regulated ubiquitin ligase, COP1, in plants.

## Author Summary

In darkness, the ubiquitin E3 ligase COP1 accumulates in the nucleus and mediates ubiquitination and degradation of positive regulators of photomorphogenesis, such as HY5. In response to light, COP1 activity is reduced to ensure proper photomorphogenic development. However, post-translational modifications that regulate COP1 activity are largely unknown. We have found that the *Arabidopsis* SUMO E3 ligase SIZ1 negatively regulates photomorphogenesis. Genetic and biochemical lines of evidence demonstrate that SIZ1-mediated SUMO modification of COP1 enhances its E3 ubiquitin ligase activity, which causes increased ubiquitination and degradation of HY5. In response to the light, sumoylation level of COP1 is decreased, which may also contributes to the reduction of COP1 activity in the light. Moreover, COP1 mediates ubiquitination and 26S proteasome-dependent degradation of SIZ1 and this feedback repression may ensure the moderate levels of COP1 activity. Our study established a post-translational regulatory modular consisting of SIZ1-mediated sumoylation and COP1-mediated ubiquitination that tightly regulate photomorphogenesis.

## Introduction

Sumoylation is a post-translational modification in which SUMO (small ubiquitin-like modifier) peptides are covalently attached to a SUMO consensus motif (ψKxE/D; ψ a large hydrophobic amino acid residue; K, the acceptor lysine; x, any amino acid; E/D, glutamate or aspartate) in target proteins through a series of biochemical steps involving activation (E1), conjugation (E2), and ligation (E3) enzymes [1, 2]. SUMO conjugation can be reversed by SUMO-specific proteases [3].

In yeast and metazoans, sumoylation has been implicated in several aspects of cellular functions, including chromatin remodeling, DNA repair, nuclear/cytoplasmic transport, transcription, and the cell cycle [4]. The PIAS [Protein inhibitors of activated STATs (signal transducer and activator of transcription)]-type SUMO E3 ligase, SIZ1 [SAP (scaffold attachment factor, acinus, PIAS), and Miz1 (Msx2-interacting zinc finger)], regulates abiotic stress responses (i.e., responses to heat, cold, drought, and salt stresses), hormone signaling (i.e., abscisic acid, salicylic acid, and auxin pathways), nutrient (i.e., phosphate, nitrogen, and copper) homeostasis, and development (i.e., flowering time and female gametophyte development) in *Arabidopsis* [5, 6].

Increasing evidence indicates that the SUMO and ubiquitin systems are tightly associated. Sumoylation antagonizes ubiquitination by competing for acceptor K residues or promotes ubiquitination by recruiting SUMO-targeted ubiquitin ligases (STUbLs) to sumoylated substrates in yeast, mammals, and plants [7, 8]. Moreover, the PIAS family of SUMO E3 ligases positively or negatively regulates ubiquitin ligase activity by SUMO modification of the SUMO-regulated ubiquitin ligases (SRUbL) in humans [9, 10]. In *Arabidopsis*, SUMO modifications regulate the protein stability of DELLA, ICE1 (inducer of CBF/DREB1 expression), ABI5 (ABA insensitive 5), MYB30, NIA1/2 (nitrate reductase), SLY1 (SLEEPY1) and SnRK1 (Snf1-related protein kinase 1) likely by antagonizing or promoting ubiquitination [11–17]. SRUbLs may also exist in plants; however, they remain to be identified.

Dark-grown seedlings have an elongated hypocotyl, closed cotyledons, and an apical hook (skotomorphogenesis). In the light, *Arabidopsis* seedlings undergo photomorphogenesis and exhibit short hypocotyls and open cotyledons with no apical hooks [18]. The ubiquitin E3 ligase COP1 (CONSTITUTIVE PHOTOMORPHOGENIC 1), a central repressor of photomorphogenesis, mediates the ubiquitination and degradation of positive regulators of photomorphogenesis, such as HY5 (ELONGATED HYPOCOTYL 5), HYH (HY5 HOMOLOGUE), LAF1 (LONG AFTER FAR-RED LIGHT 1), HFR1 (LONG HYPOCOTYL IN FAR RED 1), STH3 (SALT TOLERANCE HOMOLOG 3)/BBX2 and PIL1 (PHYTOCHROME INTERACTING FACTOR 3-LIKE1) [19–24]. SPA (SUPPRESSOR OF PHYA-105) and PIFs (PHYTOCHROME INTERACTING FACTORs) interact with COP1, and enhances its E3 ligase activity [21, 25, 26]. CSU1 (COP1 SUPPRESSOR1), a RING-finger E3 ubiquitin ligase, regulates COP1 homeostasis by ubiquitinating and degrading COP1 in darkness [27]. CSU2 and FIN219 interact with COP1, and negatively regulate its E3 ligase activity and protein level, respectively [28, 29]. In response to lights, phyA (phytochrome A), phyB, CRY1 (crytochrome 1) and CRY2, repress COP1 activity through modulating COP1-SPA1 complex [30–34], Reduced COP1 activity in the lights causes the accumulation of HY5 and the transcriptomic reprogramming of HY5 target genes [35, 36]. However, post-translational modifications that regulate COP1 activity are largely unknown.

Recent study has revealed that SUMO modification of phyB represses red light signaling, at least partly, through inhibiting interaction between phyB and PIFs [37]. In this study, we demonstrate that SIZ1 negatively regulates photomorphogenesis, at least partly, through promoting COP1 ubiquitin E3 ligase activity by SUMO modification, and that COP1 in turn mediates the ubiquitination and degradation of SIZ1. Our results reveal a novel regulatory mechanism of COP1 and SIZ1 in photomorphogenesis.

## Results

### The SUMO E3 Ligase SIZ1 Negatively Regulates Photomorphogenesis

**Fig 1:**
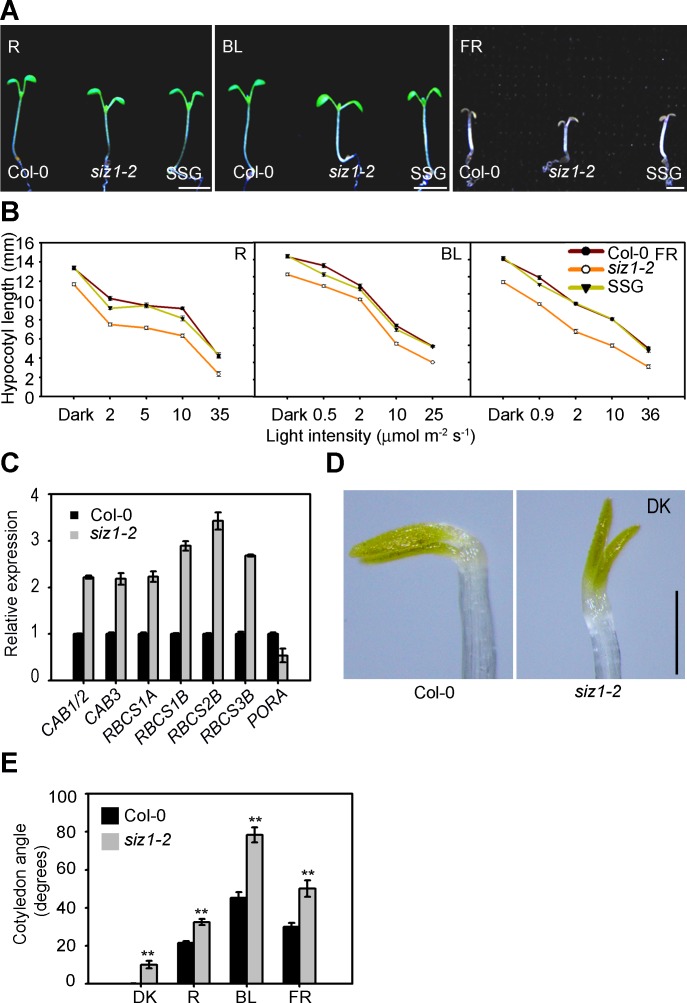
The *siz1* mutant seedlings display a short-hypocotyl phenotype. (**A**) *siz1-2* seedlings exhibit a short-hypocotyl phenotype under red (R: 10 μmol m^-2^ s^-1^), blue (BL: 14 μmol m^-2^ s^-1^), and far-red (FR: 12 μmol m^-2^ s^-1^) light conditions, and this phenotype is rescued by complementation with *SIZ1* driven by its own promoter (SSG). Bar = 2 mm. (**B**) Hypocotyl length of five-day-old Col-0, *siz1-2*, and SSG under darkness and red (R), blue (BL), and far-red (FR) light conditions at the indicated fluence rates. Data are the mean ± SE of 30 seedlings. (**C**) qRT-PCR analysis showing the enhanced responsiveness of light-responsive genes in *siz1-2* seedlings compared to those in the wild type under the dark to light transition. Five-day-old dark-grown seedlings were transferred to white light for an additional 6 h. Relative expression was normalized to that of *UBC*. Error bars indicate ± SE (n = 3). (**D**) *siz1-2* seedlings exhibit unfolded apical hooks under dark conditions (DK). Bar = 0.5 mm. (**E**) *siz1-2* seedlings show more opened cotyledon compared to Col-0 under dark (DK), red (R; 10 μmol m^-2^ s^-1^), blue (BL; 14 μmol m^-2^ s^-1^), and far-red (FR; 12 μmol m^-2^ s^-1^) light conditions. ** Student’s *t*-test indicates significant differences between the Col-0 and *siz1-2* (P ≤ 0.01).

The observation that loss-of-function *siz1* mutant seedlings showed a short-hypocotyl phenotype under white light prompted us to evaluate the light responsiveness of *siz1-2*. The *siz1-2* seedlings exhibited a short-hypocotyl phenotype under darkness and red, blue, and far-red light conditions (Fig 1A and 1B).

Expression of *ProSIZ1*:*SIZ1-GFP* in *siz1-2* plants (complemented lines referred to as SSG) [38] suppressed the short-hypocotyl phenotype of *siz1-2* under these conditions, indicating that mutation of *SIZ1* is responsible for the short-hypocotyl phenotype (Fig 1A and 1B). The short-hypocotyl phenotype of *siz1-2* was due to a reduction in cell elongation but not in cell number (S1 Fig). The expression of light-inducible genes, *CAB* (*CHLOROPHYLL A/B BINDING PROTEIN*) and *RBCS* (*RUBISCO SMALL SUBUNIT*) [39, 40], and a light-repressed gene, *PORA* (*PROTOCHLOROPHYLLIDE OXIDOREDUCTASE A*) [41], was up- and down-regulated, respectively, in *siz1-2* during the transition from darkness to light, indicating that the regulation is stronger in *siz1-2* than in wild type (Fig 1C). In addition to the short-hypocotyl phenotype, *siz1-2* seedlings had unfolded apical hooks in darkness (Fig 1D) and exhibited more opened cotyledons than did wild-type seedlings in dark and light conditions (Fig 1E). These results suggest that SIZ1 negatively regulates photomorphogenesis.

### The Short-Hypocotyl Phenotype of *siz1-2* is Due to Impaired SUMO1/2 Modification and Not Elevated SA

**Fig 2:**
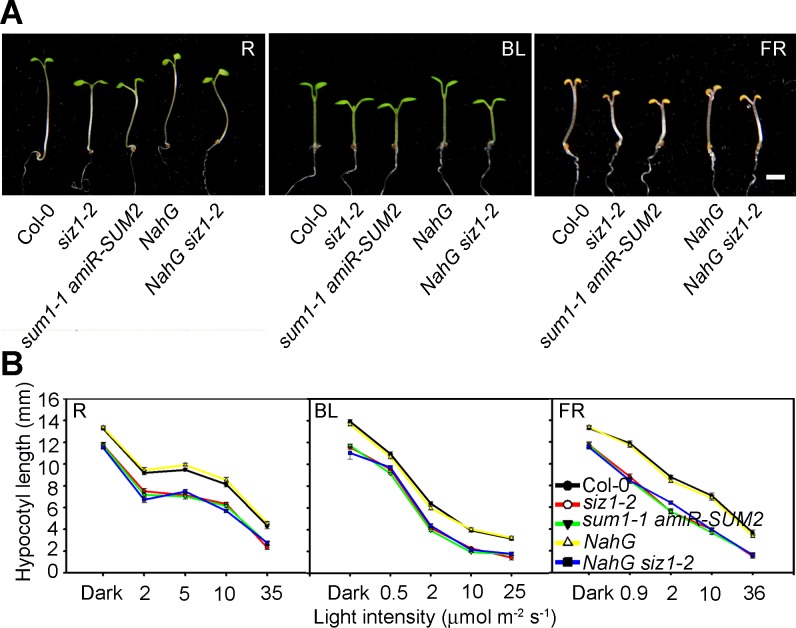
SUMO E3 ligase-mediated SUMO1/2 modification, but not SA, regulates hypocotyl elongation in response to light. (**A**) *siz1-2*, *sum1-1 amiR-SUM2*, and *NahG siz1-2* seedlings display shorter hypocotyls than the control plants Col-0 and *NahG* under red (R; 10 μmol m^-2^ s^-1^), blue (BL; 14 μmol m^-2^ s^-1^), and far-red (FR; 12 μmol m^-2^ s^-1^) light conditions. Bar = 2 mm. (**B**) Hypocotyl length of five-day-old Col-0, *siz1-2*, *sum1-1 amiR-SUM2*, *NahG*, and *NahG siz1-2* seedlings under darkness and the indicated light conditions. Data are the mean ± SE of 30 seedlings.

To determine if SIZ1-mediated SUMO1/2 modification is involved in the regulation of hypocotyl elongation, we determined the hypocotyl length of *sum1* and *sum2* double mutant under darkness and red, blue, and far-red light conditions (Fig 2).

SUMO1 and 2 have redundant functions, and the *sum1 sum2* double knockout mutant is embryo lethal [42]. Therefore, we used a viable weak allele, *sum1-1 amiR-SUM2*, in which *SUMO2* expression is down-regulated by RNAi in the *sum1-1* knockout mutant background [43]. Similar to *siz1-2*, *sum1-1 amiR-SUM2* seedlings exhibited a short-hypocotyl phenotype under dark and light conditions, suggesting that SUMO1/2 modification regulates hypocotyl elongation (Fig 2).

Elevated SA levels in *siz1-2* cause dwarfism, and the dwarf phenotype is substantially suppressed by expression of NahG, a bacterial salicylate hydroxylase [44]. To determine if the short-hypocotyl phenotype of *siz1-2* is due to increased SA levels, the hypocotyl lengths of five-day-old wild-type, *siz1-2*, *NahG*, and *NahG siz1-2* seedlings were compared under darkness and red, blue, and far-red light conditions (Fig 2). The expression of NahG did not affect hypocotyl elongation, but the *siz1-2* and *NahG siz1-2* seedlings exhibited an identical short-hypocotyl phenotype in all tested conditions, indicating that the elevated SA level in *siz1-2* did not contribute to the short-hypocotyl phenotype.

### SIZ1 Mediates SUMO Modification of COP1

**Fig 3:**
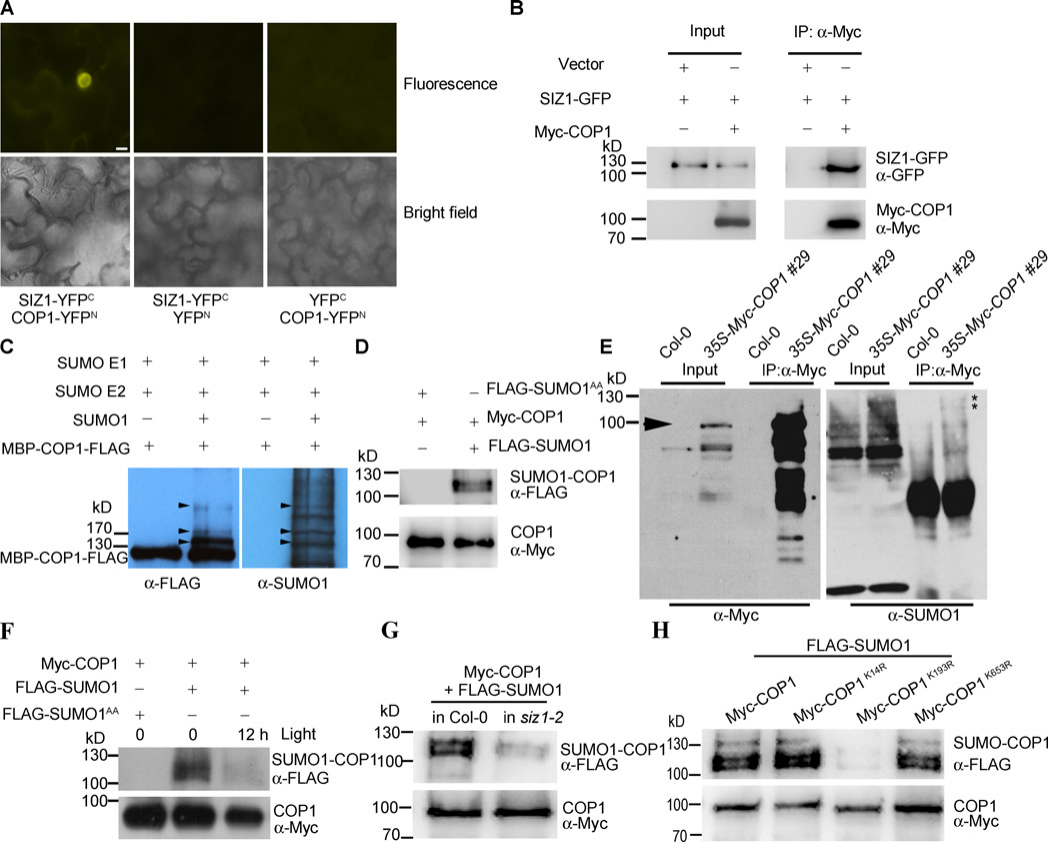
SIZ1 physically interacts with COP1, and mediates SUMO modification of COP1 at K193. (**A**) BiFC assay indicating that SIZ1-YFP^C^ interacts with COP1-YFP^N^ (left panel) in the nucleus of *N*. *benthamiana* leaf cells in the light (1 h white-light). *N*. *benthamiana* cells co-expressing SIZ1-YFP^C^ and YFP^N^ (middle panel) and YFP^C^ and COP1-YFP^N^ (right panel) were used as negative controls. Bar = 10 μm. (**B**) Co-immunoprecipitation analysis showing that SIZ1-GFP is associated with Myc-COP1. SIZ1-GFP and Myc-COP1 were transiently co-expressed in Col-0 protoplasts. Co-immunoprecipitated SIZ1-GFP was detected with anti-GFP antibody. Empty Myc vector (Vector) was used as a negative control. (**C**) *In vitro* sumoylation of COP1. Sumoylated COP1 was detected with anti-FLAG and anti-SUMO1 antibodies. Arrowheads indicate possible sumoylated COP1. (**D**) *In vivo* sumoylation of COP1. Myc-COP1 and FLAG-SUMO1 were transiently co-expressed in Col-0 protoplasts. Myc-COP1 was immunoprecipitated with anti-Myc antibody and sumoylated COP1 (SUMO1-COP1) was detected with anti-FLAG antibody. FLAG-SUMO1^AA^ was co-transformed with Myc-COP1 as a negative control. Input Myc-COP1 was detected with anti-Myc antibody. Input FLAG-SUMO1 and FLAG-SUMO1 ^AA^ were detected with anti-FLAG antibody in a separate blot, shown in S5A Fig. (**E**) Sumoylation of COP1 *in planta*. Total proteins were extracted from five-day-old dark-grown *35S-Myc-COP1* and Col-0 (control) seedlings, and anti-Myc antibody was used to immunoprecipitate Myc-COP1. Anti-SUMO1 antibody was used to determine sumoylated COP1. Input and immunoprecipitated Myc-COP1 were detected with anti-Myc antibody. Arrowhead indicates non-sumoylated COP1 band. Asterisks indicate sumoylated COP1 bands. (**F**) Light exposure reduces sumoylation levels of COP1. Myc-COP1 and FLAG-SUMO1 co-expressing *N*. *benthamiana* leaves were incubated under darkness for 12 h, and then exposed to white light (150 μmol m^-2^ s^-1^) for 12 h. The nuclear proteins were isolated at the end-of-dark (0 h) and the end-of-light (12 h) period, and the sumoylation level of COP1 was analyzed as described in (D). Input FLAG-SUMO1 and FLAG-SUMO1 ^AA^ were detected with anti-FLAG antibody in a separate blot, shown in S5B Fig. (**G**) The level of COP1 sumoylation was substantially lower in *siz1-2* than in Col-0. Myc-COP1 and FLAG-SUMO1 were transiently co-expressed in Col-0 or *siz1-2* protoplasts, and the sumoylation level of COP1 was analyzed as described in (D). (**H**) K193 is a primary sumoylation site in COP1. FLAG-SUMO1 was transiently co-expressed with Myc-COP1, Myc-COP1^K14R^, Myc-COP1^K193R^, or Myc-COP1^K653R^ in Col-0 protoplasts, and immunoprecipitation was performed as described in (D).

Since *siz1-2* seedlings exhibit a weak *cop1*-like phenotype, we examined whether SIZ1 physically interacted with COP1 to regulate its activity. To do so, we performed a bimolecular fluorescence complementation (BiFC) assay. YFP fluorescence signals were detected in the nucleus of *N*. *benthamiana* cells that coexpressed COP1-YFP^N^ (fused with the N-terminal half of YFP) and SIZ1-YFP^C^ (fused with the C-terminal half of YFP) under light (1 h light exposure) and dark conditions (Fig 3A and S2 Fig).

Consistently, SIZ1-GFP was coimmunoprecipitated with Myc-COP1 (Fig 3B). These results indicate that SIZ1 physically interacts with COP1 in the nucleus.

SUMOplot (http://www.abgent.com/sumoplot) analysis predicted the presence of three potential sumoylation motifs [45] (VK14PD, IK193ED, and WK653SD) in COP1 (S3 Fig), suggesting that COP1 may be a SUMO substrate. To test this possibility, we performed an *in vitro* sumoylation assay to determine SUMO modification of COP1 as described previously [15]. Anti-FLAG and anti-SUMO1 antibody detected slow migrating multiple bands above original COP1 protein in the reaction containing SUMO E1 (His_6_-SAE1b and His_6_-SAE2), SUMO E2 (His_6_-SCE1), and His_6_-SUMO1-GG, but not in the reaction lacking His_6_-SUMO1-GG, suggesting that COP1 is a possible SUMO substrate (Fig 3C). To further confirm COP1 is sumoylated *in vivo*, we performed an *in vivo* sumoylation analysis as described previously [38]. We co-expressed Myc-COP1 with FLAG-SUMO1 or FLAG-SUMO1^AA^ (a conjugation-deficient mutant) in *Arabidopsis* protoplasts or *N*. *benthamiana* leaves. Myc-COP1 was immunoprecipitated with anti-Myc antibody and the immunoprecipitated proteins were detected with anti-FLAG antibody. Higher molecular weight sumoylated COP1 bands were detected when Myc-COP1 and FLAG-SUMO1 were co-expressed, but not in Myc-COP1 and FLAG-SUMO1^AA^ co-expressing cells (Fig 3D and S4A Fig). Moreover, to confirm that sumoylation of COP1 occurs *in planta*, we generated an Myc-COP1 overexpression transgenic line (referred to as *35S-Myc-COP1*) and performed an *in vivo* sumoylation analysis. Anti-SUMO1 antibody detected higher molecular weight sumoylated COP1 bands (Fig 3E). Furthermore, we evaluated if sumoylation of COP1 changes in response to light. Since light exposure promotes nucleus to cytosol re-localization of COP1 [46], we monitored the sumoylation status of COP1 in the nucleus under dark (0 h) and light (12 h) conditions (Fig 3F). Under darkness, sumoylated COP1 bands were detected in the Myc-COP1 and FLAG-SUMO1 co-expressing nuclear fraction, but the sumoylation level of COP1 was substantially reduced in response to 12 h of light exposure. Taken together, these data demonstrate that COP1 is a SUMO substrate, and sumoylation level of COP1 is regulated by light.

To test if SIZ1 mediates SUMO conjugation of COP1, we cotransformed *Myc-COP1* and *FLAG-SUMO1* into *Arabidopsis* protoplasts isolated from wild-type or *siz1-2* plants, and analyzed the sumoylation status of COP1. Whereas COP1-SUMO1 conjugate was detected in the wild type, substantially lower levels were present in *siz1-2* (Fig 3G), indicating that SIZ1 facilitates the sumoylation of COP1. The lower levels of sumoylated COP1 in *siz1-2* may be due to another SUMO E3 ligase(s) that facilitates the residual SUMO modification of COP1. Alternatively, it is also possible that E1 and E2 contribute to basal levels of COP1-SUMO conjugation, since E1 and E2 alone mediate the sumoylation of COP1 *in vitro* (Fig 3C). K-to-R substitutions in sumoylation motifs block SUMO conjugation [47]. To elucidate the sumoylation motifs of COP1, SUMO conjugation of Myc-COP1^K14R^, Myc-COP1^K193R^, and Myc-COP1^K653R^ were evaluated in *Arabidopsis* protoplasts. The K193R substitution blocked COP1-SUMO1 conjugation, but K14R or K653R substitutions did not (Fig 3H). Unfortunately, we could not confirm the effect of K193R substitution *in vitro*, due to anti-FLAG antibody detected long smear bands above the original purified MBP-COP1^K193R^-FLAG, which would strongly affect subsequent *in vitro* sumoylation analysis. COP1-SUMO1 conjugation was also blocked by the K193R substitution in *N*. *benthamiana* leaves (S4B Fig). These results indicate that SIZ1 mediates sumoylation of COP1 and that K193 is critical for SUMO conjugation.

### SIZ1-Mediated Sumoylation Positively Regulates COP1 Activity

**Fig 4:**
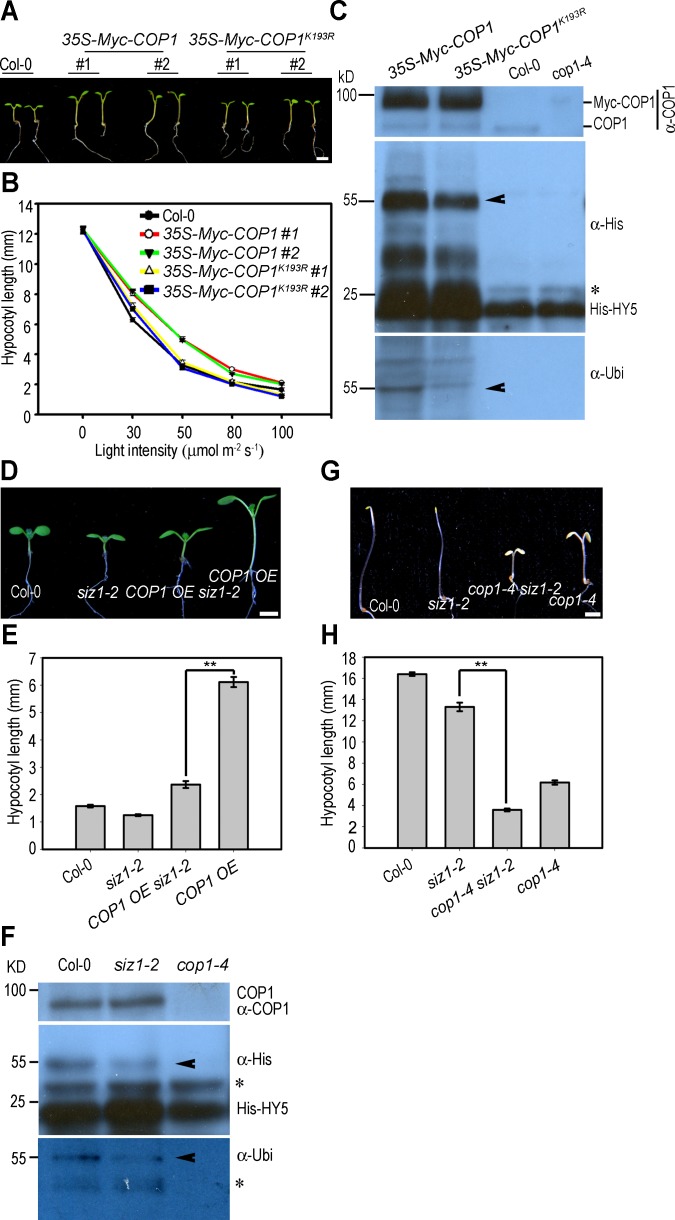
SIZ1-mediated sumoylation enhances COP1 activity. (**A**) The hypocotyls of *35S-Myc-COP1* seedlings, but not *35S-Myc-COP1*^*K193R*^, are longer than those of Col-0 seedlings under 50 μmol m^-2^ s^-1^ fluence rate of white light. Bar = 2 mm. (**B**) Hypocotyl length of five-day-old white light-grown seedlings at the indicated fluence rates. Data represent the mean ± SE (n = 30). (**C**) Total protein extracts from five-day-old dark-grown *35S-Myc-COP1*, *35S-Myc-COP1*^*K193R*^, Col-0 and *cop1-4* seedlings, and bead-conjugated 6xHis-HY5 were used as E3 ligases and substrate, respectively, to perform an *in vitro* ubiquitination assay. After reaction, the beads were washed and ubiquitinated 6xHis-HY5 were eluted for immunoblot analysis with anti-His and anti-ubiquitin antibodies. Arrowhead indicates ubiquitinated HY5. Asterisk indicates non-specific band. COP1 was detected with anti-COP1 antibody. (**D**) *siz1-2* suppresses the long-hypocotyl phenotype of *COP1 OE*. Col-0, *siz1-2*, *COP1 OE siz1-2*, and *COP1 OE* seedlings were grown under white light for 5 days. Bar = 2 mm. (**E**) Hypocotyl length of five-day-old light-grown seedlings. Data represent the mean ± SE (n = 30). ** Student’s *t*-test indicates significant differences between the *COP1 OE siz1-2* and *COP1 OE* (P ≤ 0.01). (**F**) Total protein extracts from five-day-old dark-grown Col-0 and *siz1-2* seedlings, and bead-conjugated His-HY5 were used as E3s and substrate, respectively, to perform an *in vitro* ubiquitination assay. *cop1-4* was used as a negative control. Ubiquitinated HY5 was determined using anti-His and anti-ubiquitin antibody. Arrowhead indicates ubiquitinated HY5. Asterisk indicates non-specific bands. COP1 was detected with anti-COP1 antibody. (**G**) Genetic interaction between *COP1* and *SIZ1*. Col-0, *siz1-2*, *cop1-4 siz1-2*, and *cop1-4* seedlings were grown under darkness for 5 days. Bar = 2 mm. (**H**) Hypocotyl length of five-day-old dark-grown seedlings. Data represent the mean ± SE (n = 30). ** Student’s *t*-test indicates significant differences between the *siz1-2* and *cop1-4 siz1-2* (P ≤ 0.01).

Since SIZ1 mediates SUMO modification of COP1 and *siz1-2* seedlings exhibit a weak *cop1*-like phenotype (Figs 1 and 3), we hypothesis that SIZ1-mediated SUMO modification may enhances COP1 activity. To test this possibility, we first determined the effect of sumoylation of COP1 *in planta*. Myc-COP1 and Myc-COP1^K193R^ (a non-sumoylated form) overexpressing *Arabidopsis* transgenic plants were generated, and two independent lines with similar levels of transgene expression for each construct were selected for further phenotypic analysis (S6 Fig). *35S-Myc-COP1* seedlings exhibited longer hypocotyls than did the wild type under white light conditions (Fig 4A), confirming a previous report [48].

Under 30 μmol m^-2^ s^-1^ fluence rate of white light conditions, the hypocotyls of *35S-Myc-COP1*^*K193R*^ seedlings were longer than that of the wild type, but shorter than that of *35S-Myc-COP1* (Fig 4B). Under relatively higher fluence rate of white light conditions (50 and 80 μmol m^-2^ s^-1^), while *35S-Myc-COP1* seedlings still exhibit longer hypocotyls than wild type, the hypocotyls of *35S-Myc-COP1*^*K193R*^ seedlings were of similar length as those of the wild type, suggesting that non-sumoylated Myc-COP1^K193R^ exhibits lower activity than Myc-COP1 (Fig 4A and 4B). Next, total protein extracts isolated from *35S-Myc-COP1*, *35S-Myc-COP1*^*K193R*^, Col-0 and *cop1-4* seedlings were used as E3s to perform an *in vitro* HY5 ubiquitination assay as described previously [49,50] (Fig 4C). Ubiquitination of HY5 was stronger in the reaction containing protein extracts isolated from *35S-Myc-COP1* than in that containing extracts from *35S-Myc-COP1*^*K193R*^. We did not detected ubiquitination of HY5 in the reaction containing Col-0 protein extracts due to short expose time for the blot (Fig 4C). Next, we overexpressed COP1 in *siz1-2* plants by crossing the COP1 overexpression line, *COP1 OE*, with *siz1-2* (referred to here as *COP1 OE siz1-2*), and tested if the *siz1* mutation reduces COP1 OE effect on hypocotyl elongation. Overexpression of COP1 causes a long-hypocotyl phenotype under light conditions (Fig 4D and 4E) [48]. Consistent with our hypothesis, the hypocotyl length of *COP1 OE siz1-2* was substantially shorter than that of *COP1 OE*, suggesting that COP1 activity is reduced in *siz1-2* (Fig 4D and 4E). Finally, we compared the COP1 ubiquitin E3 ligase activity of Col-0 and *siz1-2*. Total protein extracts from the wild type mediated the ubiquitination of HY5 more efficiently than did those from *siz1-2* (Fig 4F). Collectively, these results demonstrate that SIZ1-mediated SUMO conjugation promotes COP1 E3 ubiquitin ligase activity.

Furthermore, genetic interaction between *COP1* and *SIZ1* was analyzed. The hypocotyl length of *cop1-4* [51] was shorter than that of the wild type and *siz1-2* under dark and light conditions (Fig 4G and 4H and S7 Fig). The *cop1-4 siz1-2* double mutant exhibited a short-hypocotyl phenotype similar to *cop1-4* in both dark and light conditions, suggesting that SIZ1 regulates hypocotyl elongation partly through COP1 (Fig 4G and 4H and S7 Fig).

### SIZ1 Negatively Regulates HY5 Levels

**Fig 5:**
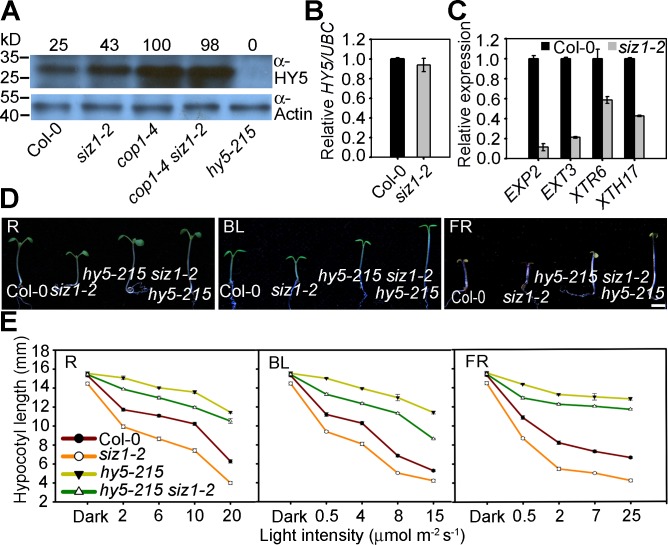
Protein abundance of HY5 is increased in *siz1-2*. (**A**) Immunoblot analysis of HY5 in Col-0, *siz1-2*, *cop1-4*, *cop1-4 siz1-2* and *hy5-215* seedlings. Total proteins were extracted from five-day-old continuous white light-grown seedlings. HY5 was detected with anti-HY5. Actin was used as a loading control and detected with anti-Actin antibody. Numbers indicate the relative protein levels of HY5. (**B**) qRT-PCR analysis of the *HY5* expression level in five-day-old continuous white light-grown Col-0 and *siz1-2* seedlings. Relative expression was normalized to that of *UBC*. Data represent the mean ± SE (n = 3). (**C**) qRT-PCR assay showing the decreased expression level of cell elongation-related genes in five-day-old continuous white light-grown *siz1-2* seedlings compared to that of Col-0. Relative expression was normalized to that of *UBC*, and data represent the mean ± SE (n = 3). (**D**) *hy5-215* suppresses the short-hypocotyl phenotype of *siz1-2* under red (R; 10 μmol m^-2^ s^-1^), blue (BL; 14 μmol m^-2^ s^-1^), and far-red (FR; 12 μmol m^-2^ s^-1^) light conditions. Bar = 2 mm. (**E**) Hypocotyl length of five-day-old seedlings under darkness and red (R), blue (BL), and far-red (FR) light conditions at the indicated fluence rates. Data represent the mean ± SE (n = 30).

SIZ1 enhances COP1 activity (Fig 4) and COP1 mediates ubiquitination and degradation of HY5 [19]. Therefore, we tested if the protein level of HY5 is up-regulated by mutation of *SIZ1*. Anti-HY5 antibody [52] revealed that endogenous HY5 was more abundant in *siz1-2* than in the wild type under light conditions (Fig 5A).

Consistent with previous report [19], higher level of HY5 was accumulated in *cop1-4*, and the HY5 level in *cop1-4* was similar to that of *cop1-4 siz1-2*, suggesting that higher level of HY5 in *siz1-2* is possibly due to reduced COP1 activity rather than increased transcription of *HY5* in *siz1-2* (Fig 5A). Consistent with this hypothesis, the level of *HY5* expression was similar in the wild type and *siz1-2* (Fig 5B). These results indicate that SIZ1 negatively regulates HY5 protein abundance. Consistent with the increased HY5 level in *siz1-2*, the expression level of the cell elongation-related direct target genes of HY5, i.e., *EXP2* (*EXPANSIN2*), *EXT3* (*EXTENSIN3*), *XTR6* (*XYLOGLUCAN ENDOTRANSGLYCOSYLASE6*), and *XTH17* (*ENDOTRANSGLUCOSYLASE/HYDROLASE17*) [36, 53], was down-regulated in *siz1-2* compared to the wild type (Fig 5C).

The loss-of-function *hy5-215* seedlings exhibited a long-hypocotyl phenotype under light conditions [54]. To identify genetic interactions between *HY5* and *SIZ1*, we generated the *hy5-215 siz1-2* double mutant through genetic crossing. The short-hypocotyl phenotype of *siz1-2* was partially suppressed by *hy5-215* under various light conditions (Fig 5D and 5E), suggesting that the accumulation of HY5 in *siz1-2* at least partly accounts for the short-hypocotyl phenotype under light conditions. In addition to HY5, COP1 also mediates the ubiquitination and degradation of HYH, LAF1, HFR1, STH3/BBX2 and PIL1 [19–24]. Thus, it is possible that the protein levels of these positive regulators of photomorphogenesis are higher in *siz1-2*, which may causes a weak *cop1-like* phenotype of *siz1-2*.

### SUMO Modification May Enhances the Transubiquitination Activity of COP1

**Fig 6:**
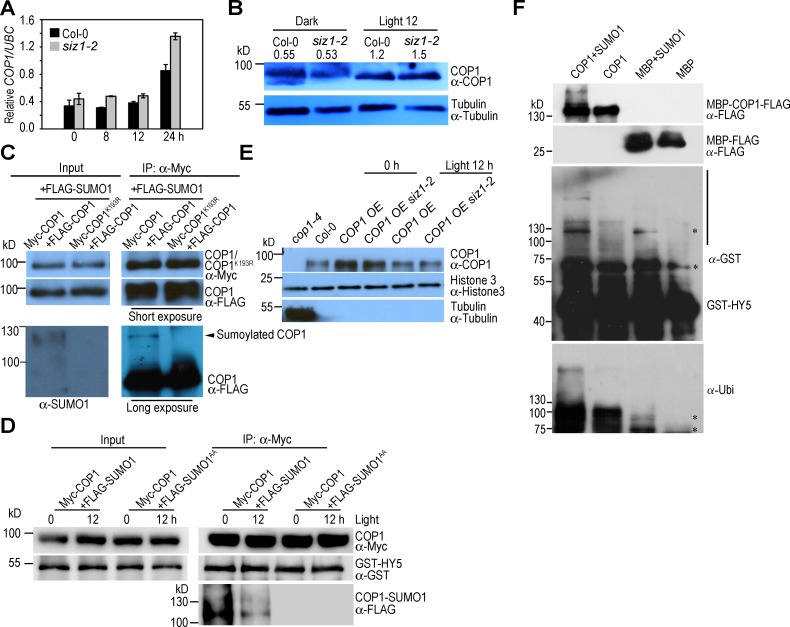
Sumoylation may enhances the transubiquitination activity of COP1. (**A**) qRT-PCR analysis of *COP1* expression in Col-0 and *siz1-2*. Five-day-old dark-grown seedlings were transferred to white light for the indicated time periods. The relative expression level of *COP1* was normalized to that of *UBC*, and data represent the mean ± SE (n = 3). (**B**) COP1 levels in Col-0 and *siz1-2* under dark and light conditions. Five-day-old dark-grown seedlings (Dark) were exposed to light for 12 h (Light 12 h). COP1 was detected with anti-COP1 antibody. Tubulin was detected with anti-Tubulin antibody as a loading control. Numbers above the blot indicate the relative level of COP1 normalized to that of Tubulin. (**C**) Analysis of the effect of SUMO modification on COP1 dimerization. FLAG-COP1 and FLAG-SUMO1 were transiently co-expressed with Myc-COP1 or Myc-COP1^K193R^ in *N*. *benthamiana* leaves. Myc-COP1 or Myc-COP1^K193R^ was immunoprecipitated (IP) with anti-Myc antibody, and FLAG-COP1 in the precipitates was detected with anti-FLAG antibody. Sumoylated COP1 was detected with anti-SUMO1 and anti-FLAG antibody (longer exposure). The level of immunoprecipitated Myc-COP1 or Myc-COP1^K193R^ was detected with anti-Myc antibody. (**D**) *In vitro* immunoprecipitation analysis of the COP1-HY5 interaction. GST-HY5 was incubated with total protein extract isolated from *N*. *benthamiana* co-expressing Myc-COP1 with FLAG-SUMO1 or FLAG-SUMO1^AA^ under dark (0 h) or light (12 h) conditions in the presence of 50 μM MG132. After 1.5 h incubation at 4°C, Myc-COP1 was immunoprecipitated with anti-Myc antibody, and co-immunoprecipitated GST-HY5 was detected with anti-GST antibody. Immunoprecipitated Myc-COP1 was quantified with anti-Myc antibody and sumoylated COP1 was detected with anti-FLAG antibody. (**E**) Nuclear fractions were isolated from *COP1 OE* and *COP1 OE siz1-2* under dark (0 h) and light (12 h white light exposure) conditions, and level of nuclear COP1 was detected with anti-COP1 antibody. Nuclear fraction of Col-0 and total protein extract of *cop1-4* was used as a control. Histone 3 and tubulin were used as nuclear and cytosol marker proteins, respectively. (**F**) *In vitro* sumoylated and non-sumoylated MBP-COP1-FLAG were used as E3 ligases to perform an *in vitro* HY5 ubiquitination assay. Bead-conjugated GST-HY5 was used as substrate. After reaction, the beads were washed and ubiquitinated GST-HY5 were eluted for immunoblot analysis with anti-GST and anti-ubiquitin antibodies. MBP-FLAG was used as a negative control. The vertical line indicates ubiquitinated HY5. Input E3s were detected with anti-FLAG antibody. Asterisks indicate non-specific bands.

SIZ1-mediated SUMO modification may regulate *COP1* expression, COP1 stability, nuclear accumulation level, interaction with other proteins and/or affects its enzymatic activity. The level of *COP1* expression was not significantly affected by the *siz1-2* mutation, indicating that SIZ1 does not regulate the transcription of *COP1* (Fig 6A).

Anti-COP1 antibody [30] revealed that endogenous COP1 protein abundance was similar in the wild type and *siz1-2*, indicating that the reduced COP1 activity in *siz1-2* was not due to reduced levels of COP1 (Fig 6B). Since COP1 functions as a homodimer [55], we examined whether SUMO modification affected COP1 dimerization. Immunoprecipitation analysis showed that Myc-COP1 and Myc-COP1^K193R^ (a non-sumoylated form) immunoprecipitated the same amount of FLAG-COP1, indicating that sumoylation of COP1 did not significantly affect COP1 dimerization (Fig 6C). Next, we examined if SUMO modification enhanced the COP1-HY5 interaction using *in vitro* co-immunoprecipitation assays. Sumoylation of COP1 did not enhance the substrate accessibility of COP1 under dark and light conditions (Fig 6D). Moreover, the *siz1* mutation did not affect the level of nuclear COP1 under dark and light conditions (Fig 6E). Finally, we evaluated if SUMO conjugation regulates transubiquitination acitivity of COP1. *In vitro* sumoylated and non-sumoylated MBP-COP1-FLAG were used as E3s to perform an *in vitro* HY5 ubiquitination assay. Anti-GST and anti-ubiquitin antibodies detected higher level of ubiquitinated proteins in the reaction containing sumoylated COP1 than did non-sumoylated COP1 (Fig 6F), suggesting that SIZ1-mediated SUMO modification may enhances transubiquitination activity of COP1.

### COP1 Mediates the Ubiquitination and Degradation of SIZ1

**Fig 7:**
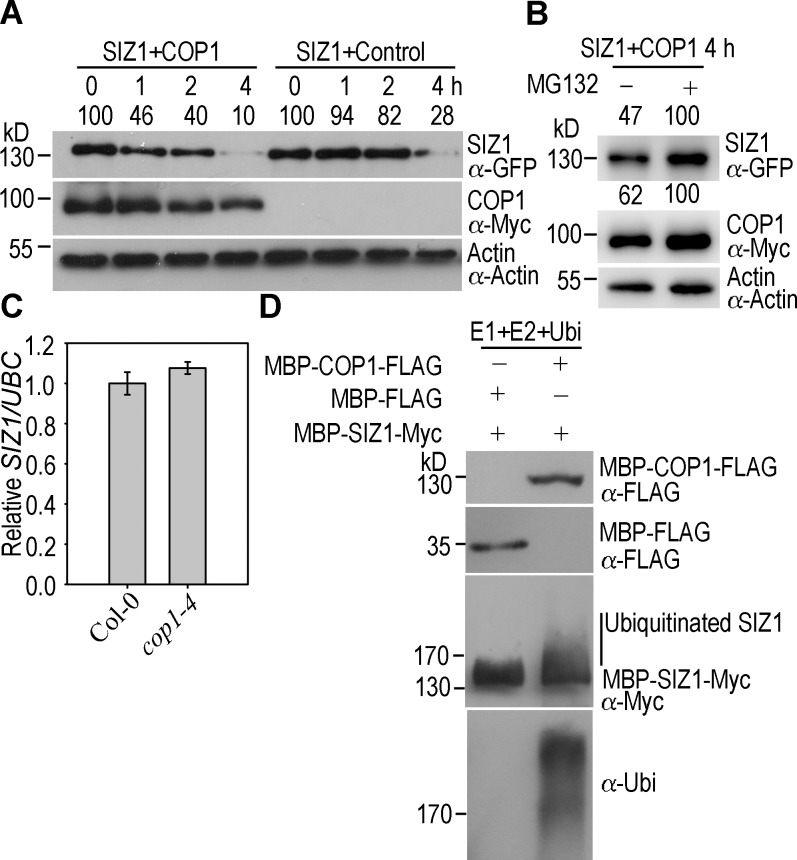
COP1 promotes the ubiquitination and degradation of SIZ1. (**A**) COP1 enhances the degradation of SIZ1. Total proteins extracted from Myc-COP1 expressing or non-transformed (control) *N*. *benthamiana* leaves were mixed with total proteins extracted from SIZ1-GFP expressing *N*. *benthamiana* leaves, and incubated for the indicated periods at 4°C under dark condition. SIZ1-GFP and Myc-COP1 protein levels were analyzed with the anti-GFP and anti-Myc antibody, respectively. Actin was used as a loading control and detected with anti-Actin antibody. Numbers indicate the relative protein levels of SIZ1. (**B**) MG132 inhibits the degradation of SIZ1-GFP. Total proteins extracted from Myc-COP1 or SIZ1-GFP expressing leaves were mixed together, and treated with or without 50 μM MG132 for an additional 4 h at 4°C under dark condition. The levels of SIZ1-GFP and Myc-COP1 were analyzed as describe above. Actin was used as a loading control and detected with anti-Actin antibody. Numbers indicate the relative protein levels of SIZ1 and COP1. (**C**) qRT-PCR analysis of the transcription level of *SIZ1* in Col-0 and *cop1-4*. Seedlings were grown under white light for 5 days. Relative expression was normalized to that of *UBC*. Data indicate the mean ± SE (n = 3). (**D**) *In vitro* ubiquitination of SIZ1 by COP1. MBP-COP1-FLAG and bead-conjugated MBP-SIZ1-Myc were used as E3 ligases and substrate, respectively, to perform an *in vitro* ubiquitination assay. MBP-FLAG was used as a negative control. Input E3s were detected with anti-FLAG antibody. After reaction, the beads were washed and ubiquitinated MBP-SIZ1-Myc was eluted for immunoblot analyses with anti-Myc and anti-ubiquitin antibodies. The vertical line indicates ubiquitinated SIZ1 proteins.

Although the biological functions of SIZ1 have been extensively characterized, the mechanism that regulates SIZ1 activity is largely unknown. Our finding that COP1 interacted with SIZ1 (Fig 3A and 3B) prompted us to determine if COP1 mediates the ubiquitination and degradation of SIZ1. To determine if COP1 promotes degradation of SIZ1, we analyzed the decay kinetics of SIZ1-GFP in the presence or absence of Myc-COP1. The degradation of SIZ1 was more rapid in the presence of Myc-COP1 (Fig 7A).

Moreover, the COP1-promoted SIZ1 degradation was inhibited by MG132, a 26S proteasome inhibitor (Fig 7B, upper panel). In agreement with a previous report [21], MG132 also repressed the degradation of COP1 (Fig 7B, middle panel). *SIZ1* expression was not affected in *cop1-4*, indicating that COP1 does not regulate SIZ1 transcription (Fig 7C). Next, we evaluated if COP1 mediates the ubiquitination of SIZ1. MBP-COP1-FLAG was used as the E3 ligase in an *in vitro* SIZ1 ubiquitination assay. Anti-Myc and anti-ubiquitin antibody revealed that COP1 facilitates ubiquitination of SIZ1 *in vitro* (Fig 7D). These results suggest that COP1 negatively regulates SIZ1 protein stability through ubiquitination and subsequent 26S proteasome-dependent degradation.

## Discussion

COP1 protein stability is regulated by CSU1-mediated ubiquitination [27]. However, how the COP1 E3 ligase activity is regulated by post-translational modifications remains largely unknown. Our genetic and biochemical analyses revealed that COP1 activity is enhanced by SIZ1-mediated SUMO modification.

### The Role of SUMO Modifications in Photomorphogenesis

Loss-of-function *siz1-2* and *sum1-1 amiR-SUM2* seedlings exhibited a short-hypocotyl phenotype under dark and light conditions (Figs 1 and 2), suggesting that SIZ1-mediated SUMO1/2 modification negatively regulates photomorphogenesis. SIZ1 physically interacts with COP1 and facilitates its SUMO modification (Fig 3). COP1 exhibits higher activity compared to that of non-sumoylated COP1, COP1^K193R^, and the COP1 activity was attenuated in *siz1-2* (Fig 4). The reduced COP1 activity in *siz1* resulted in higher levels of HY5, stronger down-regulation of cell elongation-related HY5 direct target genes (Fig 5A and 5C). These results strongly suggest that SIZ1-mediated SUMO modification enhances COP1 activity; thus, COP1 is a plant SUMO-regulated ubiquitin ligases (SRUbL).

The hypocotyl length of *cop1-4 siz1-2* was slightly shorter than that of *cop1-4* (Fig 4G and 4H and S7 Fig), suggesting that SIZ1 regulates hypocotyl elongation through at least two pathways, a COP1-dependent and -independent pathway. Recently, it has been shown that SUMO modification of phyB negatively regulate photomorphogenesis under red light [37]. OTS1 (OVERLY TOLERANT TO SALT1) mediate desumoylation of phyB, but whether the SUMO modification is facilitated by SIZ1 remains to be determined. However, it should be noted that *cop1-4* is a weak allele, which expresses a partially functional truncated COP1 protein (1–282 aa, which contains the K193 sumoylation motif) [51]. Therefore, it is also possible that the partial COP1 activity in *cop1-4* is further attenuated in *cop1-4 siz1-2*.

### Regulation of COP1 Activity by SUMO Modifications

It has been demonstrated that COP1 is regulated by different mechanisms: (1) light, FIN219 and cold induces nuclear-to-cytoplasmic export of COP1, but ethylene enhances nuclear retention of COP1 in the light [29, 56–58]; (2) CSU1, FIN219 and heat shocks reduce protein abundance of COP1 [27, 29, 59]; (3) SPA positively regulates COP1 activity may through enhancing substrate recruitment [21, 25]; (4) phyA, phyB, CRY1 and CRY2 repress COP1 activity by affecting COP1-SPA complex in the lights [30–34]; (5) PIF1 not only enhances substrate recruitment, but also increases autoubiquitination and transubiquitination activities of COP1 [26]. The present study reveals that SIZ1-mediated sumoylation does not regulate COP1 protein abundance, dimerization of COP1, or nuclear-to-cytoplasmic translocation (Fig 6). SUMO modification possibly increased transubiquitination activity of COP1 *in vitro* (Fig 6F), but did not affect substrate recruitment (Fig 6D). At the molecular level, sumoylation affects protein-protein interactions and target protein conformation [60, 61]. We speculate that the enhanced transubiquitination activity of COP1 by sumoylation possibly due to conformational change of COP1 or increased COP1-E2 interaction. Recent study has demonstrated that CSU2 negatively regulates COP1 activity through their coiled-coil domains [28]. SUMO modification site, K193, is located in the coiled-coil domain of COP1. Thus, it is also possible that the sumoylation inhibits interaction between CSU2 and COP1 *in vivo*, but remain to be elucidated in the near future.

It has been shown that a certain amount of COP1 is present in the nucleus even under prolonged light exposure, and plays a critical role in regulating development [27, 51, 58, 62–64]. Interestingly, prolonged light exposure reduced sumoylation levels of nuclear-localized COP1 (Fig 3F), which results in decreased COP1 activity. Thus, we hypothesize that light-induced reduction of COP1-SUMO levels is required for the maintenance of moderate COP1 activity under light conditions to ensure the tight regulation of photomorphogenesis. The reduced sumoylation level of COP1 under light conditions may be due to decreased SIZ1-mediated sumoylation and/or increased SUMO protease(s)-mediated desumoylation, but the details of the mechanisms by which light regulates the balance between sumoylation and desumoylation remain to be elucidated.

**Fig 8:**
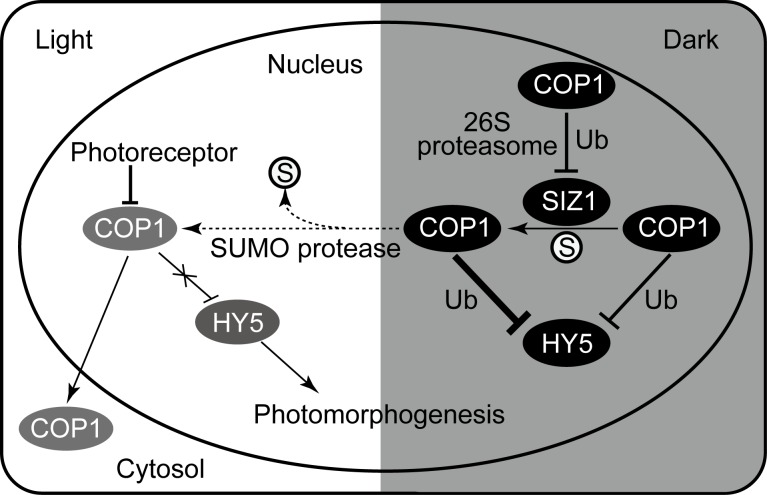
Proposed model of how SIZ1 and COP1 regulate photomorphogenesis. Under darkness, COP1 mediates the ubiquitination and degradation of HY5 (thinner T-bar). SIZ1-mediated SUMO modification enhances COP1 ubiquitin E3 ligase activity toward HY5 (thicker T-bar), but COP1 in turn promotes ubiquitination and 26S proteasome-mediated SIZ1 degradation. Under light, COP1 activity is repressed by photoreceptors and nuclear exclusion. Light exposure reduces the sumoylation level of COP1, which may also contribute to the repression of COP1 activity. SUMO protease possibly mediates the desumoylation of COP1 in response to light. T-bars indicate negative regulation and arrows indicate positive regulation. Dotted line indicates hypothetical regulation. S and Ub indicate SUMO and Ubiquitin, respectively.

Collectively, our study demonstrates that SIZ1-mediated sumoylation negatively regulates photomorphogenesis, at least partly, through enhancing COP1 activity. Interestingly, COP1 in turn mediates the ubiquitination and 26S proteasome-dependent degradation of SIZ1 (Fig 8). This feedback repression of SIZ1 activity by COP1 may reflect the requirement of tightly regulated COP1 activity for proper photomorphogenic development.

## Materials and Methods

### Plant Materials

Seeds of wild type, *siz1-2* (Salk_065397), *cop1-4*, *COP1 OE*, *hy5-215*, *sum1-1 amiR-SUM2* [43], SSG [38], *NahG*, and *NahG siz1-2* [44] were in the Columbia ecotype. *cop1-4 siz1-2*, *hy5-215 siz1-2*, and *COP1 OE siz1-2* were generated by genetic crossing and the double mutants were identified as described in previous reports [51, 54]. To generate *35S-Myc-COP1* and *35S-Myc-COP1*^*K193R*^, pBI121-Myc-COP1 and pBI121-Myc-COP1^K193R^ were transformed into Col-0 by the *Agrobacterium*-mediated floral dip method [65], respectively, and homozygous T3 plants were used.

### Plant Growth Conditions and Phenotypic Analysis

After surface sterilization, seeds were sown on Murashige and Skoog growth medium (1/4× Murashige and Skoog basal salts, 1% sucrose, and 0.75% agar). After 3 days of incubation at 4°C in darkness, the seeds were exposed to 6 h of white light to induce germination, and then transferred to light chambers containing red, blue, or far-red light emitting diodes at the indicated fluence rates. White light (100 μmol m^-2^ s^-1^ unless indicated otherwise) was provided using white fluorescent lamps. To analyze hypocotyl length, cotyledon angle, and hypocotyl cell length and cell numbers, seedlings were photographed with a camera (Canon) or a cool CCD camera coupled to an Olympus BX53 microscope and the images were analyzed with NIH ImageJ software (http://rsbweb.nih.gov/ij/).

### RNA Extraction and Quantitative Real-Time Reverse Transcription-PCR

Total RNA was extracted from seedlings with TRIZOL reagent (RNAiso Plus, TaKaRa) and reverse transcribed using a RevertAid First Strand cDNA Synthesis Kit (Thermo Scientific). qRT-PCR was then performed with SYBR Premix ExTaq (TaKaRa) according to the manufacturer’s instructions. Three biological replicates were performed. The relative expression level of each gene was normalized to that of *Ubiquitin-Conjugation Enzyme* (*UBC*). Primer sequences are listed in S1 Table.

### BiFC Assay

COP1-YFP^N^ and SIZ1-YFP^C^ plasmids together with the proper control plasmids (empty YFP^N^ or YFP^C^ vector plasmids) were transformed into *Agrobacterium tumefaciens* strain GV3101 and infiltrated into *Nicotiana benthamiana* leaves as previously described [66]. After incubation at 22°C for 3 days under 16 h white light (100 μmol m^-2^ s^-1^)/8 h darkness, YFP fluorescence was detected with a fluorescence microscope (Olympus BX53) under darkness or after 1 h white light (100 μmol m^-2^ s^-1^) exposure.

### Immunoprecipitation Assays

For co-immunoprecipitation of COP1 and SIZ1, Myc-COP1 and SIZ1-GFP were separately expressed in Col-0 protoplasts to avoid degradation of SIZ1 by COP1. Immunoprecipitation was carried out using a mixture of COP1 and SIZ1 protein extracts. The mixture of protein extracts was immunoprecipitated with anti-Myc-conjugated agarose beads (Sigma, F-2426) and co-immunoprecipitated SIZ1-GFP proteins were detected with anti-GFP antibody (Clontech, 632375).

To analyze the sumoylation effect in the interaction between HY5 and COP1, 1 μg of GST-HY5 protein purified from *E*. *coli* [52], as bait, was incubated with the protein extracts isolated from *N*. *benthamiana* co-expressing Myc-COP1 with FLAG-SUMO1 or FLAG-SUMO1^AA^. The mixture of protein extracts was immunoprecipitated with anti-Myc-conjugated agarose beads (Sigma, F-2426) and co-immunoprecipitated GST-HY5 proteins were detected with anti-GST antibody (Abcam, ab19256).

### *In vivo* Sumoylation Assay

To determine the sumoylation status of COP1, proteins were extracted in a buffer composed of 50 mM Tris-Cl (pH 7.4), 150 mM NaCl, 1 mM EDTA (pH 8.0), 1 mM DTT, 20 mM NEM, 1% TritonX-100, and 1× complete protease inhibitor mixture (Roche, 04693159001). Then, the protein extracts were immunoprecipitated with anti-Myc-conjugated agarose beads (Sigma, F-2426) for 3 h. Next, the beads were washed with protein extraction buffer four times, and the immunoprecipitated proteins were eluted with 2× SDS loading buffer for immunoblot analysis. The sumoylated form of COP1 was identified with anti-FLAG antibody (Sigma, F3165) or anti-SUMO1 antibody (Abcam, ab5316).

### *In vitro* Sumoylation Assay

An *in vitro* sumoylation assay was performed as described previously [15] with minor modifications. Briefly, 50 ng of His_6_-AtSAE1b, 50 ng of His_6_-AtSAE2, 50 ng of His_6_-AtSCE1, 8 μg of His_6_-AtSUMO1-GG, and 100 ng of MBP-COP1-FLAG were incubated in 30 μl of reaction buffer (20 mM HEPES pH7.5, 5 mM MgCl_2_, 2 mM ATP) for 3 h at 30°C. Sumoylated MBP-COP1-FLAG was detected with anti-FLAG (Sigma, F3165) and anti-SUMO1 antibody (Abcam, Ab5316).

### *In vitro* Ubiquitination Assay

An *in vitro* ubiquitination assay was performed as described previously [50] with minor modifications. E3s, recombinant wheat E1, 500 ng purified E2, 5 μg ubiquitin, and 100 ng substrates were incubated in 30 μl of reaction buffer (50 mM Tris-Cl (pH 7.4), 10 mM MgCl_2_, 5 mM ATP, and 2 mM DTT) for 3 h at 30°C. The substrates were detected with anti-His (Sigma, H9658), anti-GST (Abcam, ab19256), or anti-Myc (Sigma, M4439) antibody, and the ubiquitination was determined with anti-ubiquitin (Sigma, U5379) antibody.

### Nuclear Protein Extraction

Nuclear protein extraction was carried out with the CelLytic PN Extraction Kit (Sigma, CELLYTPN1) as described previous [62].

### Plasmid Construction

To generate pSPYNE-COP1-YFP^N^, full-length *COP1* cDNA without the termination codon was amplified with gene-specific primers COP1-F-*Spe*I/COP1nt-R-*Xho*I. The *COP1* cDNA was inserted in-frame at the *Spe*I/*Xho*I sites of the pSPYNE-35S vector [66].

To generate pSPYCE-SIZ1-YFP^c^, full-length *SIZ1* cDNA without the termination codon was amplified with gene-specific primers SIZ1-F-*Xma*I/SIZ1-R-*Spe*I and ligated into the pBluescript vector. pBluescript-SIZ1 was digested with *Sma*I and *Spe*I, and the *SIZ1* cDNA was inserted in-frame at the *Hpa*I/*Spe*I sites of the pSPYCE-35S vector [66].

To generate pCambia1302-SIZ1-GFP, pBluescript-SIZ1 was digested with *Sma*I and *Spe*I. The pCambia1302 vector was digested with *Nco*I, to generate blunt ends, and then with *Spe*I. The insert was then ligated into the pCambia1302 vector.

To generate pMAL-C2-MBP-COP1-FLAG, full-length cDNA of *COP1* without the termination codon was amplified with gene-specific primers COP1-5’-*Sma*I/COP1nt-3’-*Xho*I, and ligated into the pBluescript vector. pBluescript-COP1 was digested with *Sma*I and *Xho*I, and inserted in-frame at the *Sma*I and *Xho*I sites of the pMAL-C2-MBP-FLAG vector.

To generate pMAL-C2-MBP-SIZ1-Myc, full-length cDNA of *SIZ1* without the termination codon was amplified with gene-specific primers SIZ1-F-*Xba*I/SIZ1-R-*Cla*I, and ligated into the pBluescript vector. pBluescript-SIZ1 was digested with *Xba*I and *Cla*I, and inserted in-frame at the *Xba*I and *Cla*I sites of the pMAL-C2-MBP-MYC vector.

To generate p326-Myc-COP1, the full-length cDNA of *COP1* was amplified with gene-specific primers COP1-F-*Hind*III/COP1-R-*Xho*I, and ligated into the pBluescript vector. pBluescript-COP1 was digested with *Hind*III and *Xho*I, and inserted in-frame at the *Hind*III and *Xho*I sites of the p326-35S-nMyc vector.

pBluescript-COP1 was used as template with primer pairs COP1 K14R-F/COP1 K14R-R, COP1 K193R-F/COP1 K193R-R, and COP1 K653R-F/COP1 K653R-R, to generate pBluescript-COP1^K14R^, pBluscript-COP1^K193R^, and pBluescript-COP1^K653R^, respectively. pBluescript-COP1^K14R^, pBluescript-COP1^K193R^, and pBluescript-COP1^K653R^ were digested with *Hind*III and *Xho*I, and inserted in-frame at the *Hind*III and *Xho*I sites of the p326-35S-nMyc vector to generate p326-35S-Myc-COP1^K14R^, p326-Myc-COP1^K193R^, and p326-Myc-COP1^K653R^, respectively. p326-Myc-COP1 and p326-Myc-COP1 ^K193R^ were digested with *Spe*I and *Xho*I, and the digested products were inserted into the *Spe*I and *Xho*I sites of the pBI121-35S-nMyc vector to generate pBI121-Myc-COP1 and pBI121-Myc-COP1^K193R^, respectively.

To generate p326-FLAG-SUMO1 and pBI121-FLAG-SUMO1, *SUMO1* cDNA was amplified with gene-specific primers SUMO-F-*Xba*I and SUMO-R-*Xho*I, and inserted into the *Xba*I and *Xho*I sites of the p326-35S-nFLAG and pBI121-35S-nFLAG vector. To generate p326-FLAG-SUMO1^AA^ and pBI121-FLAG-SUMO1^AA^, *SUMO1* cDNA was amplified with gene-specific primers SUMO-F-*Xba*I and SUMO^AA^-R-*Xho*I, and inserted into the *Xba*I and *Xho*I sites of the p326-35S-nFLAG and pBI121-35S-nFLAG vector. All primer sequences are listed in S1 Table.

## Supporting Information

S1 Fig The Short-hypocotyl phenotype of *siz1-2* is associated with reduced cell length but not cell number. Hypocotyl length, hypocotyl cell length, and hypocotyl cell number of five-day-old Col-0 and *siz1-2* seedlings grown under red (a), blue (b), and far-red (c) light. Error bars represent ± SE (n = 30). Double asterisks indicate significant differences between Col-0 and *siz1-2* (P ≤ 0.01), as determined by Student’s *t*-test analysis. Fluence rates of lights were 10 μmol m^-2^ s^-1^ for red light, 14 μmol m^-2^ s^-1^ for blue light, and 12 μmol m^-2^ s^-1^ for far-red light.
(TIF)

S2 Fig COP1 interacts with SIZ1 under darkness. Bimolecular fluorescence complementation (BiFC) assay indicating that SIZ1-YFP^C^ interacts with COP1-YFP^N^ (left panel) in the nucleus of *N*. *benthamiana* leaf cells under darkness. *N*. *benthamiana* cells co-expressing SIZ1-YFP^C^ and YFP^N^ (middle panel) and YFP^C^ and COP1-YFP^N^ (right panel) were used as negative controls. Bar = 10 μm.
(TIF)

S3 Fig Potential sumoylation sites in COP1. (**A**) COP1 contains an N-terminal ring finger zinc-binding (RING) motif, a coiled-coil domain (Coil), and C-terminal multiple WD40 repeat domain (WD40). Potential sumoylation sites K14, K193, and K653 are indicated. (**B**) Amino acid sequence of COP1. Potential sumoylation sites are highlighted in red.
(TIF)

S4 Fig COP1 is a SUMO substrate and K193 is a primary sumoylation site. (**A**) COP1 is sumoylated in *N*. *benthamiana*. Myc-COP1 and FLAG-SUMO1 were transiently co-expressed in *N*. *benthamiana* leaves. Total protein was immunoprecipitated with anti-Myc antibody, and SUMO1-COP1 conjugate was detected with anti-FLAG antibody. FLAG-SUMO1^AA^ and Myc-COP1 were co-transformed into *N*. *benthamiana* leaves as a negative control. Input Myc-COP1 was analyzed with anti-Myc antibody. (**B**) The K193R substitution blocks COP1 sumoylation in *N*. *benthamiana*. FLAG-SUMO1 was co-expressed with Myc-COP1 or Myc-COP1^K193R^ in *N*. *benthamiana* leaves. Myc-COP1 was immunoprecipitated with anti-Myc antibody and SUMO1 conjugates were determined with anti-FLAG antibody. Input Myc-COP1 or Myc-COP1^K193R^ was analyzed with anti-Myc antibody.
(TIF)

S5 Fig Expression level of free FLAG-SUMO1/FLAG-SUMO1^AA^ in Fig 3D and 3F. Myc-COP1 and FLAG-SUMO1/FLAG-SUMO1^AA^ were transiently co-expressed in Col-0 protoplasts (A; for Fig 3D) or *N*. *benthamiana* leaves (B; for Fig 3F). Total proteins were extracted and the input FLAG-SUMO1 and FLAG-SUMO1 ^AA^ were detected with anti-FLAG antibody.
(TIF)

S6 Fig Expression level of COP1 in *35S-Myc-COP1* and *35S-Myc-COP1*^*K193R*^ transgenic plants. (**A**) qRT-PCR analysis of the *COP1* expression level in five-day-old continuous white light-grown Col-0, *35S-Myc-COP1*, and *35S-Myc-COP1*^*K193R*^ seedlings. Relative expression was normalized to that of *UBC*. Data represent the mean ± SE (n = 3). (**B**) Immunoblot analysis of Myc-COP1 in Col-0, *35S-Myc-COP1* and *35S-Myc-COP1*^*K193R*^ seedlings. Total proteins were extracted from five-day-old continuous white light-grown seedlings. COP1 was detected with anti-COP1 antibody. Actin was used as a loading control and detected with anti-Actin antibody.
(TIF)

S7 Fig Genetic interaction between *SIZ1* and *COP1*. Five-day-old light-grown seedlings (left panel) and the hypocotyl lengths (right panel) of Col-0, *siz1-2*, *cop1-4 siz1-2*, and *cop1-4* seedlings grown under (a) red (R; 10 μmol m^-2^ s^-1^), (b) blue (BL; 14 μmol m^-2^ s^-1^), and (c) far-red (FR; 12 μmol m^-2^ s^-1^) light. Data represent the mean ± SE (n = 30). Bar = 2 mm. ** Student’s *t*-test indicates significant differences between the *siz1-2* and *cop1-4 siz1-2* (P ≤ 0.01).
(TIF)

S1 Table List of primers used in this study. (DOC)
