# Supplementary figures and images for: An Arabidopsis SUMO E3 Ligase, SIZ1, Negatively Regulates Photomorphogenesis by Promoting COP1 Activity
Source: PLoS Genet. 2016 Apr 29;12(4):e1006016. doi: 10.1371/journal.pgen.1006016 (PMC4851335; doi:10.1371/journal.pgen.1006016)

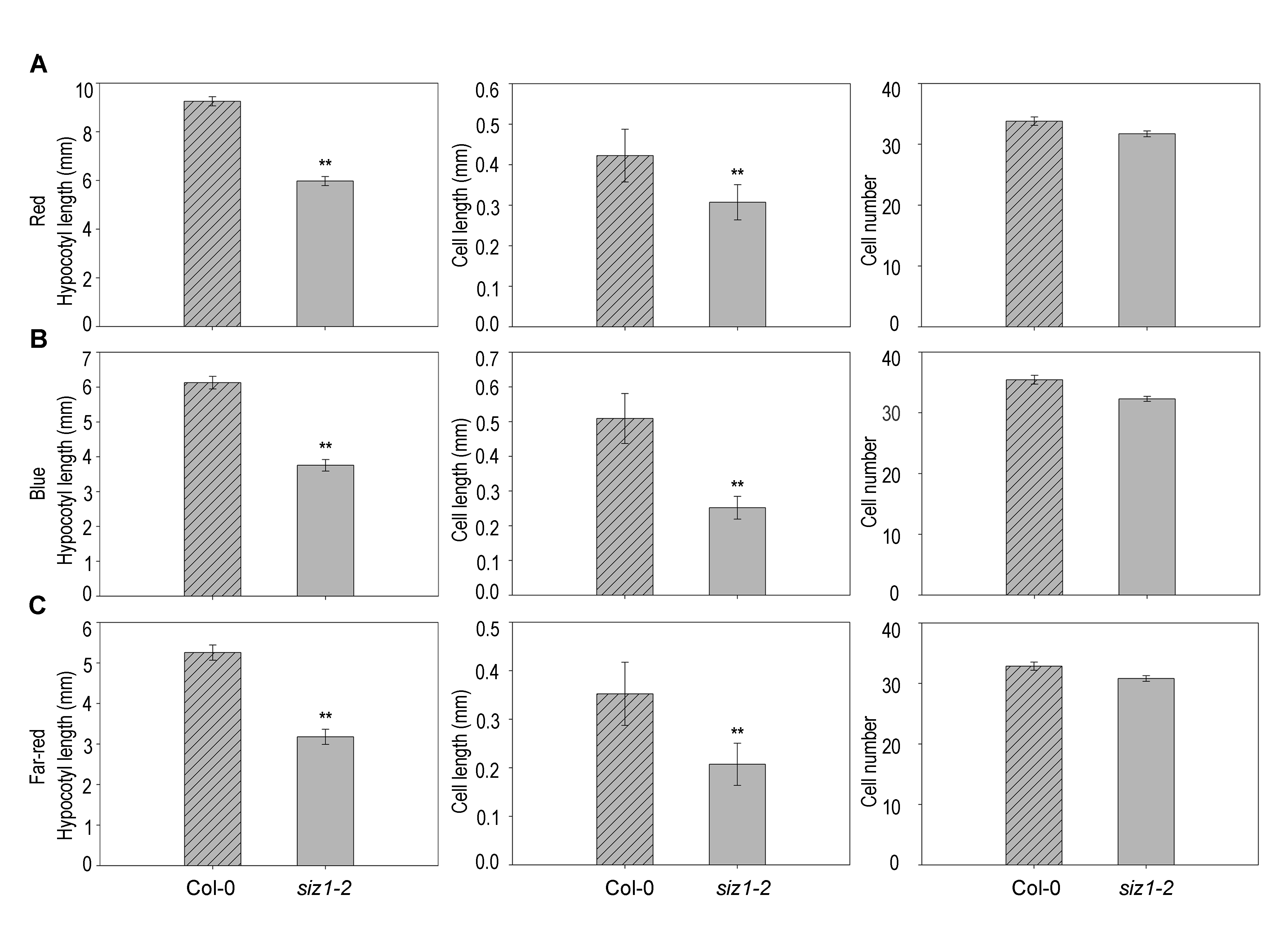

Supplement: S1 Fig — Hypocotyl length, hypocotyl cell length, and hypocotyl cell number of five-day-old Col-0 and siz1-2 seedlings grown under red (a), blue (b), and far-red (c) light. Error bars represent ± SE (n = 30). Double asterisks indicate significant differences between Col-0 and siz1-2 (P ≤ 0.01), as determined by Student’s t-test analysis. Fluence rates of lights were 10 μmol m-2 s-1 for red light, 14 μmol m-2 s-1 for blue light, and 12 μmol m-2 s-1 for far-red light. (TIF) [file pgen.1006016.s001.tif]

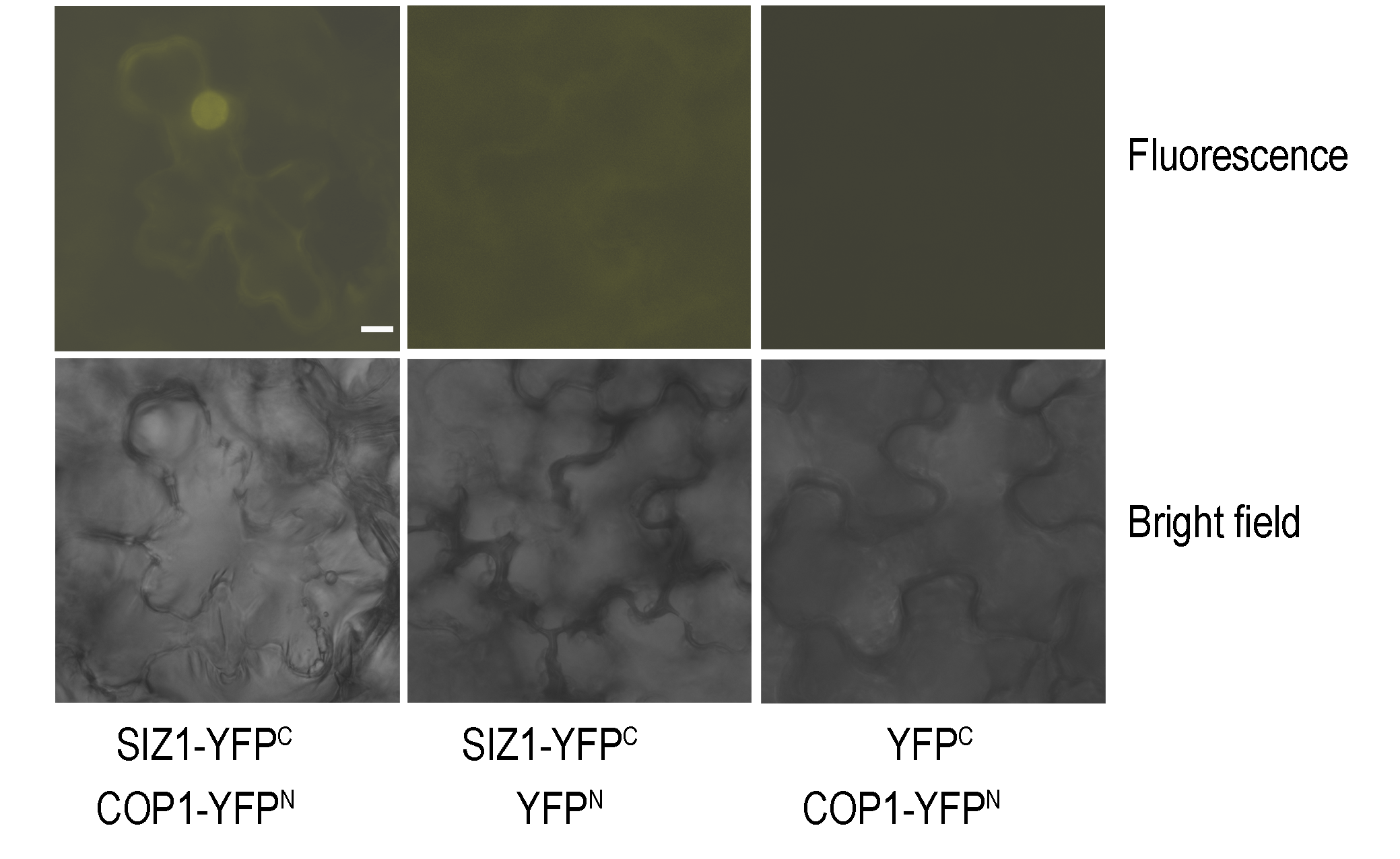

Supplement: S2 Fig — Bimolecular fluorescence complementation (BiFC) assay indicating that SIZ1-YFPC interacts with COP1-YFPN (left panel) in the nucleus of N. benthamiana leaf cells under darkness. N. benthamiana cells co-expressing SIZ1-YFPC and YFPN (middle panel) and YFPC and COP1-YFPN (right panel) were used as negative controls. Bar = 10 μm. (TIF) [file pgen.1006016.s002.tif]

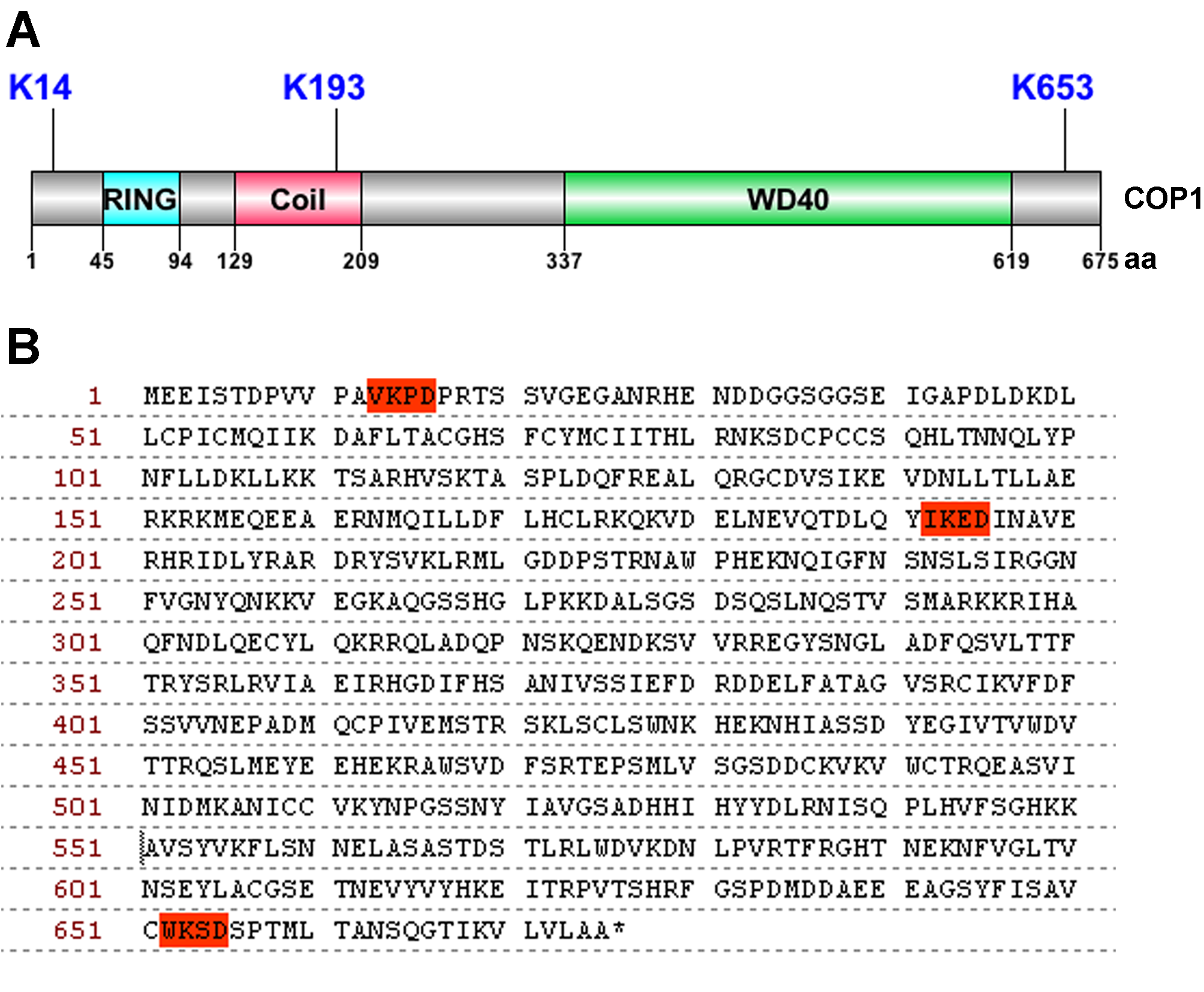

Supplement: S3 Fig — (A) COP1 contains an N-terminal ring finger zinc-binding (RING) motif, a coiled-coil domain (Coil), and C-terminal multiple WD40 repeat domain (WD40). Potential sumoylation sites K14, K193, and K653 are indicated. (B) Amino acid sequence of COP1. Potential sumoylation sites are highlighted in red. (TIF) [file pgen.1006016.s003.tif]

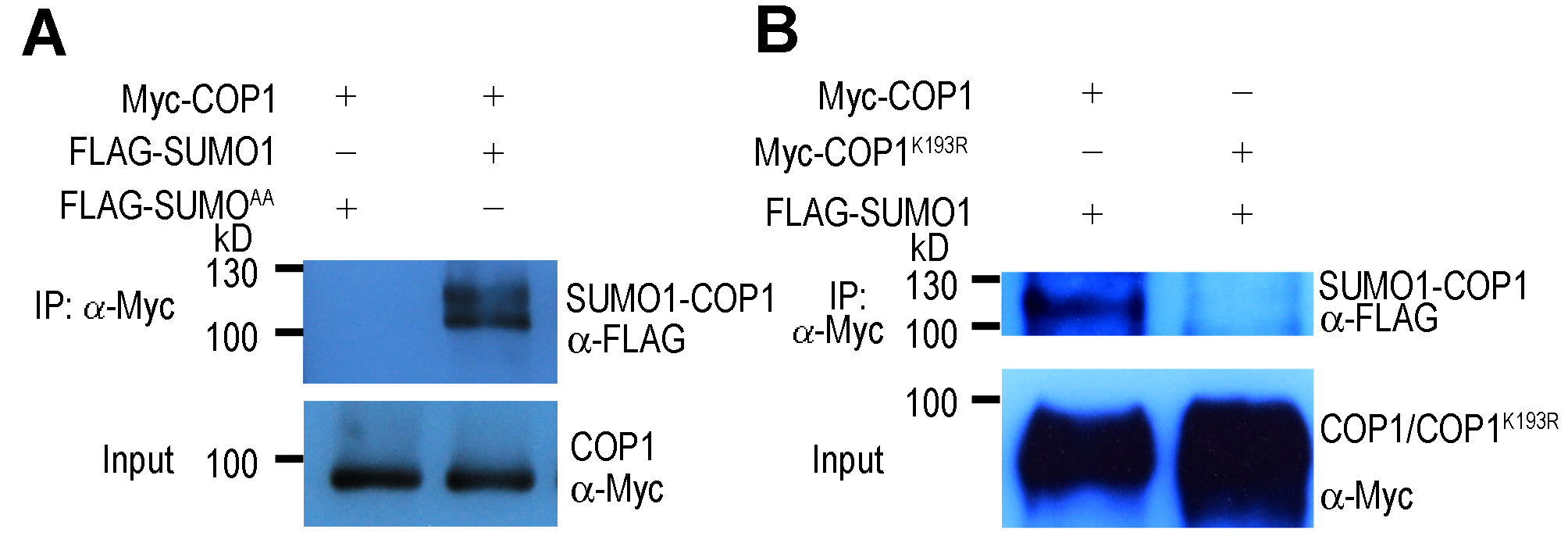

Supplement: S4 Fig — (A) COP1 is sumoylated in N. benthamiana. Myc-COP1 and FLAG-SUMO1 were transiently co-expressed in N. benthamiana leaves. Total protein was immunoprecipitated with anti-Myc antibody, and SUMO1-COP1 conjugate was detected with anti-FLAG antibody. FLAG-SUMO1AA and Myc-COP1 were co-transformed into N. benthamiana leaves as a negative control. Input Myc-COP1 was analyzed with anti-Myc antibody. (B) The K193R substitution blocks COP1 sumoylation in N. benthamiana. FLAG-SUMO1 was co-expressed with Myc-COP1 or Myc-COP1K193R in N. benthamiana leaves. Myc-COP1 was immunoprecipitated with anti-Myc antibody and SUMO1 conjugates were determined with anti-FLAG antibody. Input Myc-COP1 or Myc-COP1K193R was analyzed with anti-Myc antibody. (TIF) [file pgen.1006016.s004.tif]

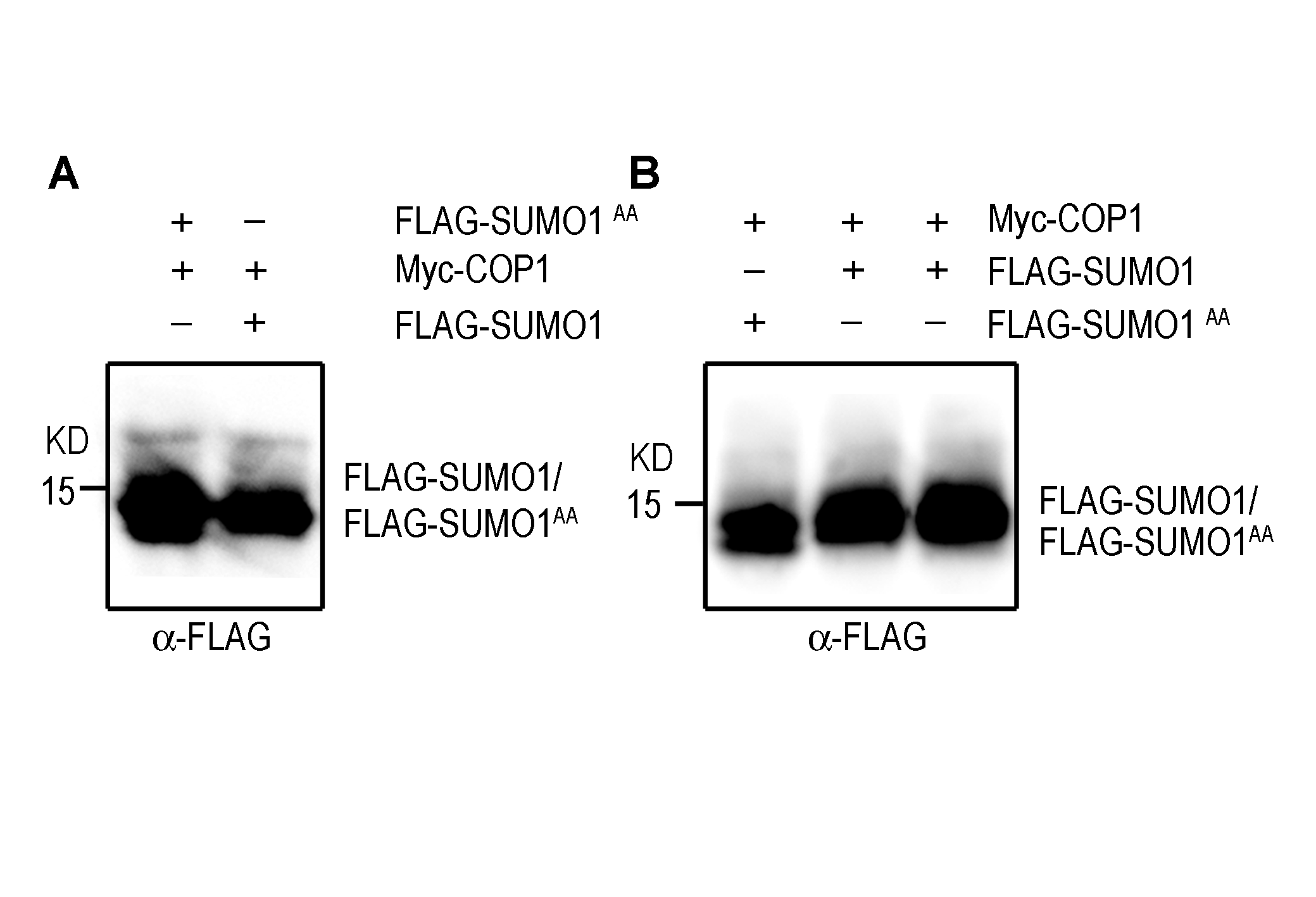

Supplement: S5 Fig — Myc-COP1 and FLAG-SUMO1/FLAG-SUMO1AA were transiently co-expressed in Col-0 protoplasts (A; for Fig 3D) or N. benthamiana leaves (B; for Fig 3F). Total proteins were extracted and the input FLAG-SUMO1 and FLAG-SUMO1 AA were detected with anti-FLAG antibody. (TIF) [file pgen.1006016.s005.tif]

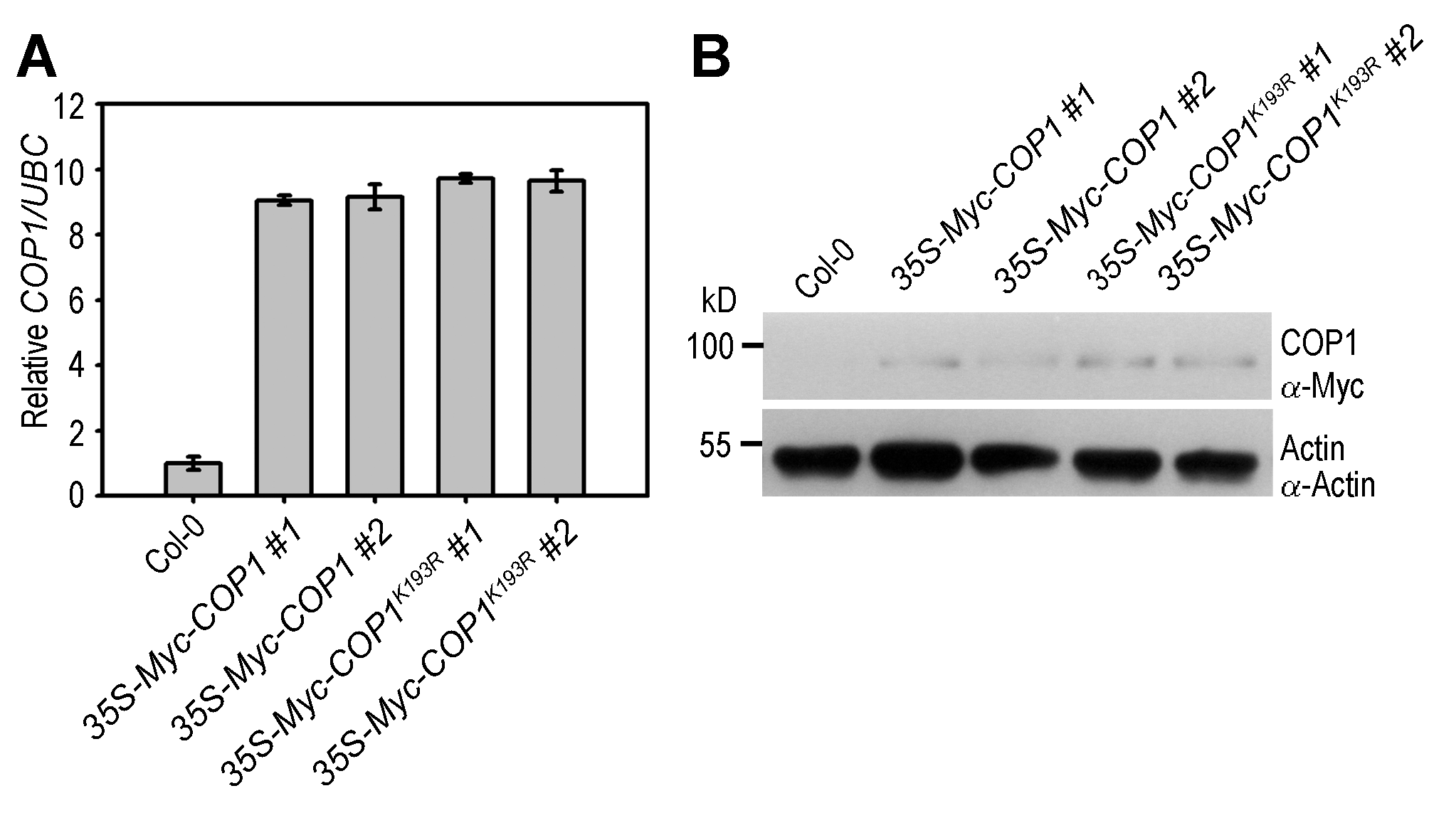

Supplement: S6 Fig — (A) qRT-PCR analysis of the COP1 expression level in five-day-old continuous white light-grown Col-0, 35S-Myc-COP1, and 35S-Myc-COP1K193R seedlings. Relative expression was normalized to that of UBC. Data represent the mean ± SE (n = 3). (B) Immunoblot analysis of Myc-COP1 in Col-0, 35S-Myc-COP1 and 35S-Myc-COP1K193R seedlings. Total proteins were extracted from five-day-old continuous white light-grown seedlings. COP1 was detected with anti-COP1 antibody. Actin was used as a loading control and detected with anti-Actin antibody. (TIF) [file pgen.1006016.s006.tif]

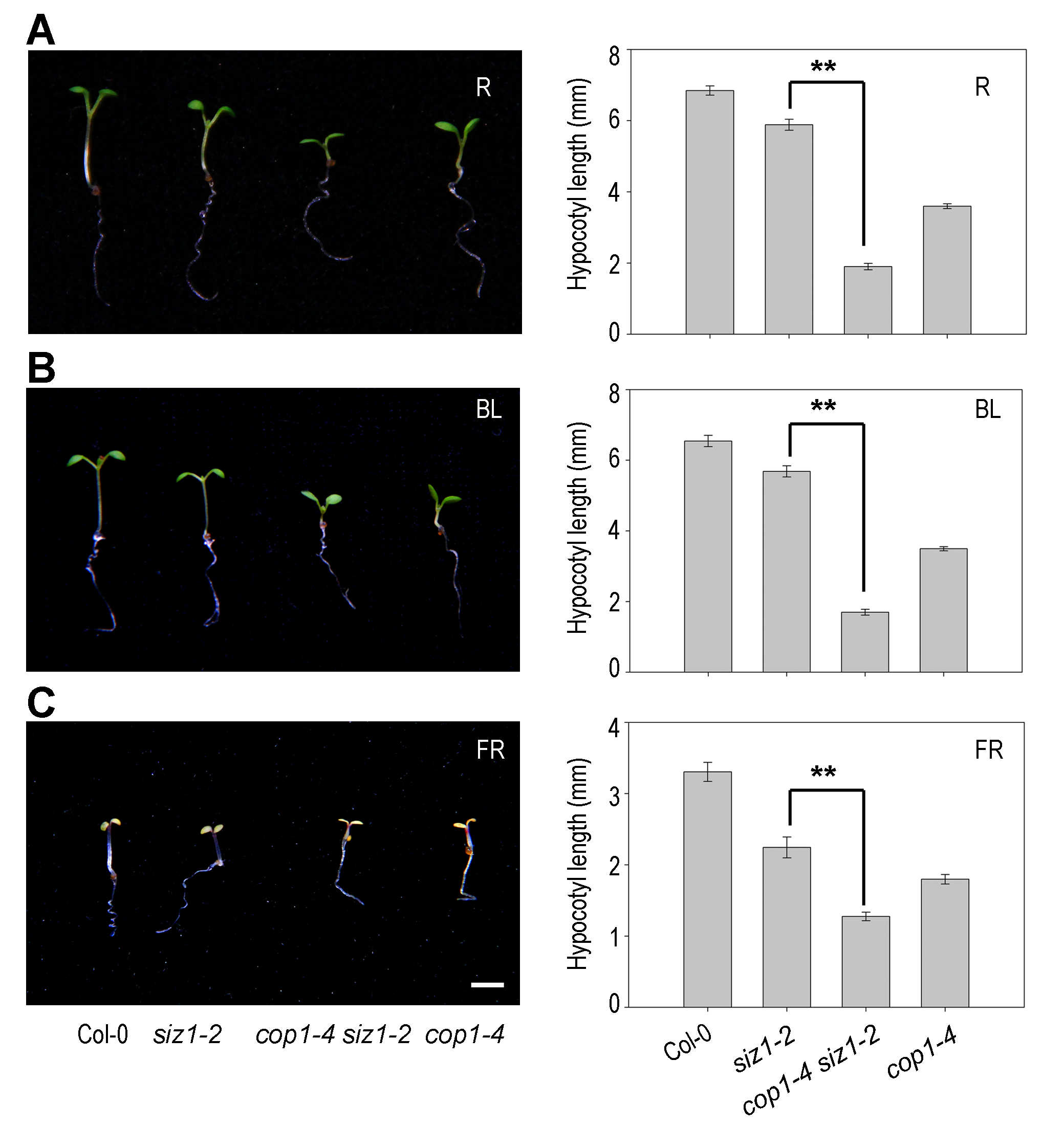

Supplement: S7 Fig — Five-day-old light-grown seedlings (left panel) and the hypocotyl lengths (right panel) of Col-0, siz1-2, cop1-4 siz1-2, and cop1-4 seedlings grown under (a) red (R; 10 μmol m-2 s-1), (b) blue (BL; 14 μmol m-2 s-1), and (c) far-red (FR; 12 μmol m-2 s-1) light. Data represent the mean ± SE (n = 30). Bar = 2 mm. ** Student’s t-test indicates significant differences between the siz1-2 and cop1-4 siz1-2 (P ≤ 0.01). (TIF) [file pgen.1006016.s007.tif]
