# Supplementary material for: An Arabidopsis SUMO E3 Ligase, SIZ1, Negatively Regulates Photomorphogenesis by Promoting COP1 Activity
Source: PLoS Genet. 2016 Apr 29;12(4):e1006016. doi: 10.1371/journal.pgen.1006016 (PMC4851335; doi:10.1371/journal.pgen.1006016)
Supplement: S1 Table — (DOC) [file pgen.1006016.s008.doc]

**S1 Table. List of Primers Used in This Study**

For cloning

| Oligo name | Sequence (5’-3’) | Purpose |
| --- | --- | --- |
| COP1-F-*Hind*III  COP1-R-*Xho*I | CCC aagcttATGGAAGAGATTTCGACGGAT  CCG ctcgagTCACGCAGCGAGTACCAGA | p326-Myc-COP1 |
| COP1-F-*Spe*I  COP1nt-R-*Xho*I | actagtATGGAAGAGATTTCGACGG  ctcgagCGCAGCGAGTACCAGAAC | pSPYNE-35S:COP1-YFP^N^ |
| COP1-5’-*Sma*I  COP1nt-3’-*Xho*I | TCCcccgggATGGAAGAGATTTCGACGGAT  CCGctcgagCCGCAGCGAGTACCAGAACT | pMAL-C2-MBP-COP1-FLAG |
| COP1 K14R-F  COP1 K14R-R | TCGGGTCAGGTCTCACCGCTGGAACAACC  TCCAGCGGTGAGACCTGACCCGAGAACATC | Site-directed mutagenesis |
| COP1 K193R-F  COP1 K193R-R | TATATCTTCTCTAATATACTGGAGATC  CAGTATATTAGAGAAGATATAAATGCC | Site-directed mutagenesis |
| COP1 K653R-F  COP1 K653R-R | GACTATCACTCCTCCAGCAAACCGCAC  GGTTTGCTGGAGGAGTGATAGTCCCACG | Site-directed mutagenesis |
| SUMO-F-*Xba*I  SUMO-R-*Xho*I  SUMO^AA^-R-*Xho*I | GC tctagaATGTCTGCAAACCAGGAGGA  CCG ctcgagTCAGCCACCAGTCTGATGGAG  CCG ctcgagTCAGGCAGCAGTCTGATGGAG | p326-FLAG-SUMO1  pBI121-FLAG-SUMO1  p326-FLAG-SUMO1^AA^ pBI121-FLAG-SUMO1^AA^ |
| SIZ1-F-*Xma*I  SIZ1-R-*Spe*I | cccgggATGGATTTGGAAGCTAATTG  actagtCTCAGAATCCGAGTCAAT | pSPYCE-35S:SIZ1-YFP^C^  pCambia1302-SIZ1-GFP |
| SIZ1-F- *Xba*I  SIZ1-R- *Cla*I | GCtctagaATGGATTTGGAAGCTAATTGTAA  CCatcgtaCCTCAGAATCCGAGTCAATGG | pMAL-C2-MBP-SIZ1-Myc |

For qRT-PCR

| Gene | AGI code | Oligo name | Sequence (5’-3’) |
| --- | --- | --- | --- |
| *COP1* | AT2G32950 | COP1-Q-F  COP1-Q-R | TCTGCGTCCACAGATAGCAC  AGGAACCTGCCTCTTCCTCT |
| *SIZ1* | AT5G60410 | SIZ1-Q-F  SIZ1-Q-R | TTTTGGGTTACAGTGGCACA  ACACTCTGCATTGTGCTTGC |
| *HY5* | AT5G11260 | HY5-Q-F  HY5-Q-R | GTTTGGAGGAGAAGCTGTCG  TCTTGCTTGCTGAGCTGAAA |
| *CAB1/2* | AT1G29930  AT1G29920 | CAB1/2-Q-F  CHS1/2-Q-R | GCCTCAACAATGGCTCTCTC  GCTTGGCAACAGTCTTCCTC |
| *CAB3* | AT1G29910 | CAB3-Q-F  CAB3-Q-R | CTTTCAGCTGATCCCGAGAC  CCCACCTGCTGTGGATAACT |
| *RBCS1A* | AT1G67090 | RBCS1A -Q-F  RBCS1A -Q-R | ACCTTATCCGCAACAAGTGG  TTGTCCAGTACCGTCCATCA |
| *RBCS1B* | AT5G38430 | RBCS1B -Q-F  RBCS1B -Q-R | CCACCAATCGGAAAGAAGAA  ATCCATTTGTTGCGGAGAAG |
| *RBCS2B* | AT5G38420 | RBCS2B -Q-F  RBCS2B -Q-R | CCACCAATCGGAAAGAAGAA  TACACAAATCCGTGCTCCAA |
| *RBCS3B* | AT5G38410 | RBCS3B -Q-F  RBCS3B -Q-R | GCCACCAATTGGAAAGAAGA  ATCCACTTGTTGCGGAGAAG |
| *PORA* | AT5G54190 | PORA-Q-F  PORA-Q-R | GATGCAAGAGGGAACAGAGC  AGTTGAAGTCGCGATTGCTT |
| *EXP2* | AT5G05290 | EXP2-Q-F  EXP2-Q-R | TGTCCTCCAAACTTTGCCTTAG  CCTTATCCCTCCACCTTTCTCA |
| *EXT3* | AT1G21310 | EXT3-Q-F  EXT3-Q-R | GGGTCTCCAATGGCCTCTTTA  CGTAGTGCTTCTTAGGTGGTGGT |
| *XTR6* | AT4G25810 | XTR6-Q-F  XTR6-Q-R | CTGGGATTTACAGGGAGAAGG  TGATGGCAGTAGCACAGAAGAA |
| *XTH17* | AT1G65310 | XTH17-Q-F  XTH17-Q-R | CTTGGGAAACATAAGTGGTCATC  TGTCTAGTTGGGAACGGGACT |
| *UBC21* | AT5G25760 | UBC-Q-F  UBC-Q-R | TTCAAATGGACCGCTCTTATC  GCTCAGGATGAGCCATCAAT |

For genotyping

| Mutant | Marker | Marker Sequence (5’-3’) | Genotyping |
| --- | --- | --- | --- |
| *siz1-2* | LP  RP  LBa1 | GAGCTGAAGCATCTGGTTTTG  CACGACAGATGAAGCATTGTG  TGGTTCACGTAGTGGGCCATCG | LP/RP 913 bp  RP/LB a1 ~500 bp |
